# Brominated Compounds from Marine Sponges of the Genus *Aplysina* and a Compilation of Their 13C NMR Spectral Data

**DOI:** 10.3390/md9112316

**Published:** 2011-11-10

**Authors:** Narlize Silva Lira, Ricardo Carneiro Montes, Josean Fechine Tavares, Marcelo Sobral da Silva, Emidio V. L. da Cunha, Petronio Filgueiras de Athayde-Filho, Luis Cezar Rodrigues, Celidarque da Silva Dias, Jose Maria Barbosa-Filho

**Affiliations:** 1 Laboratory of Pharmaceutical Technology, Federal University of Paraiba, Joao Pessoa 58051-900, PB, Brazil; E-Mails: narlizelira@yahoo.com.br (N.S.L.); ricsony_79@yahoo.com.br (R.C.M.); josean@ltf.ufpb.br (J.F.T.); marcelosobral@ltf.ufpb.br (M.S.d.S.); athayde-filho@quimica.ufpb.br (P.F.d.A.-F.); lcezar@ltf.ufpb.br (L.C.R.); 2 Department of Pharmacy, State University of Paraiba, Campina Grande 58100-000, PB, Brazil; E-Mail: emidio@ltf.ufpb.br

**Keywords:** *Aplysina*, marine sponges, halogenated substances, ^13^C NMR, review

## Abstract

*Aplysina* is the best representative genus of the family Aplysinidae. Halogenated substances are its main class of metabolites. These substances contribute greatly to the chemotaxonomy and characterization of the sponges belonging to this genus. Due to their pharmacological activities, these alkaloids are of special interest. The chemistry of halogenated substances and of the alkaloids has long been extensively studied in terrestrial organisms, while the number of marine organisms studied has just started to increase in the last decades. This review describes 101 halogenated substances from 14 species of *Aplysina* from different parts of the world. These substances can be divided into the following classes: bromotyramines (A), cavernicolins (B), hydroverongiaquinols (C), bromotyrosineketals (D), bromotyrosine lactone derivatives (E), oxazolidones (F), spiroisoxazolines (G), verongiabenzenoids (H), verongiaquinols (I), and dibromocyclohexadienes (J). A compilation of their ^13^C NMR data is also part of the review. For this purpose 138 references were consulted.

## 1. Introduction

Marine sponges have been known and used by mankind since antiquity. They were included in the first classification of living organisms, written in 350 BC by Aristotle in Greece. At first thought to be plants, their animal nature was only recognized by the end of the XVIII century. However, great naturalists of the time such as Lamarck, Linnaeus and Cuvier classified them as Zoophytes. The elevation of the Porifera to the level of phylum was suggested by Huxley in 1875 and by Sollas in 1884, and was only accepted at the beginning of the XX century [1].

Sponges belong to the phylum Porifera and are the most primitive of multicelled animals, having existed for roughly 700–800 million years. They have a very simple physiology of construction. They are aquatic organisms growing mostly in temperate salt waters but may also be found in fresh water. When reaching adult form, they are found in solid substrates in places that allow adequate conditions for their growth. Some, when in their primary states, may be mobile [2–4]. They are easily found in all marine environments, from the intertidal zones to the ocean depths of 8500 m in tropical and polar seas. Despite their wide distribution in terms of different oceans and depths, the rocky non-polluted coastline areas show greater populations of sponges which are also known for being rich in secondary metabolites [5–9].

The sponges are filtering animals, which utilize flagellate cells called coenocytes for promoting the circulation of the water through a system of canals existing in this phylum only called aquifer system, around which their body is built. This water flow brings organic particles and microorganisms which are filtered and eaten [10]. Of all the known sponges, only 1% grow in fresh water [11].

There are basically three classes of sponges, Calcarea (5 orders and 24 families), Desmospongiae (15 orders and 92 families) and Hexactinellida (6 orders and 20 families). So far, about 15,000 species of sponges have been described, their diversity however is believed to be much bigger than this [4,12]. Being sessile simple organisms, they evolved chemical defense mechanisms to protect themselves against predators and competitors, as well as against infectious microorganisms. Studies show that secondary metabolites in sponges carry out a crucial role in their survival in the marine ecosystem [13,14].

Because of their potential for the production of new substances of pharmacological interest, sponges have been one of the most chemically studied organisms. In the past 20 years, hundreds of substances have been isolated from them and many of those substances have already been identified, and present interesting biological and pharmacological (such as antibacterial, anticoagulant, antifungal, antimalarial, antituberculosis, antiviral, immunosuppressive and neuro-suppressor) activities [15–23]. The main reported activities for the *Aplysina* genus are antibacterial, antiyeast, antifungal, antiviral, cytotoxic and hyperglycemic activities, which can be seen in Table 1.

The pioneer investigative work in the field of sponge chemistry published by Bergmann and Feeney in the beginning of the 1950s led to the discovery of *Cryptotethya crypta* bioactive nucleosides spongothymidine and spongouridine [21]. These nucleosides were the basis for the synthesis of Ara-C, the first marine derivative anticancer agent, and antiviral drug Ara-A [22]. Today, Ara-C is used in the routine treatment of patients suffering from leukemia and lymphomas. One of its derivatives was also approved for use in patients with cancer of the pancreas, lungs and breast [23].

### The Genus Aplysina

The genus *Aplysina*, formerly known as *Verongia* and reclassified to *Aplysina*, is one of the richest in terms of secondary metabolites, described in 14 species of the family Aplysinidae, there are 2 species from the Mediterranean Sea, 8 from the Caribbean, 3 from the Pacific Coast of Mexico and 15 in the Brazilian coast. Of the above species, 8 have only been recently identified. From the Mediterranean Sea, the two described species of the genus *Aplysina* are: *A. aerophoba* (Schmidt, 1862) and *A. cavernicola* (Vacelet, 1959). From the Caribbean, among others we find *A. fistularis insularis*, *A. fistularis* form *fulva*, *A. archeri*, *A. cauliformis* and *A. Lacunosa* [33].

Like other genera of the order Verongida, *Aplysina* stands out for its unique biochemical characteristics. They show low terpene content, and possess a moderately high percentage of sterols, mostly within the aplystan skeleton. They also produce a significant series of brominated derivatives of tyrosine metabolites considered peculiar to species of this order. The sponges of this order are also known for their high phenotypic variability [34].

Marine organisms produce a cocktail of halogenated metabolites with potential commercial value. The structures found in these compounds go from linear chain acyclic, to complex polycyclic molecules [35,36]. The research of halogenated metabolites has been more focused on marine algae than on sea sponges [37–41]. Though many compounds have been discovered recently, many sponges species are poorly screened and the need for new drugs keeps this field open.

In a previous paper our research group evaluated crude algae, sponge extracts and chemically determined molecules from Northeastern Brazil [42–48] with database survey [49–62].

In this paper we review halogenated substances from the genus *Aplysina*. A compilation of the ^13^C NMR spectral data of the selected natural products is also provided. This type of genus and species investigation is helpful in the identification and capture of halogenated substances from the genus.

## 2. Methodology

An extensive bibliographic review was carried out to identify studies of halogenated substances isolated from the genus *Aplysina*. The present review covers the period of 1967 thru 2010. The search was performed using the following databases: NAPRALERT (Natural Products Alert at the University of Illinois, Chicago), Chemical Abstracts, and the Brazilian online scientific literature search system called “Periodical CAPES” (Coordination for the Improvement of Graduate Level Personnel).

Tables 2 and 3 respectively show the halogenated substance distribution in the genus *Aplysina*, and the basic skeletons of those substances. Table 4 shows the different substituents for the diverse classes of halogenated substances. Table 5 describes the position of the substituents for the 101 substances isolated from each species. Finally Tables 6–14 show a compilation of ^13^C NMR data of the substances.

Although the isoxazoline alkaloids are the group with more ^13^C NMR data, some chiral centers of this group continue with an undefined stereochemistry due to the incompatibility of using X-ray crystallography techniques, possessing sometimes non-crystalline characteristic [98]. Some positions with ^13^C NMR data had to be revised because there were mistakes in the numbering of the carbon skeleton in the attribution of values of some positions in this group of alkaloids.

## 3. Discussion

The genus *Aplysina* belongs to the order Verongida, sponges with a wide variety of metabolites. Sterols [110,111], carotenoids [112], amino acids [113] and rare fatty acids [114] have all been isolated from this order. However, the peculiarity of this order is from the ecological and medicinal points of view, in that great production of halogenated substances originates from the metabolism of amino acids such as phenylalanine and tyrosine. The halogenated substances found in the marine sponges of the genus *Aplysina* can be classified as: (A) Bromotyramines, (B) Cavernicolins, (C) Hydroverongiaquinols, (D) Bromotyrosineketals, (E) Bromotyrosine lactone derivatives, (F) Oxazolidones, (G) Spiroisoxazolines, (H) Verongiabenzenoids, (I) Verongiaquinols and (J) Dibromociclohexadiens.

### 3.1. Chemotaxonomy Importance of *Aplysina* Sponges

Although in the past, it was suspected that bromotyrosine compounds were not present in Brazilian *Aplysina* species [69], nowadays numerous studies have shown the presence of these chemical biomarkers, not only in Brazilian species, but in almost all the Verongida order.

In order to classify the large number of halogenated compounds reviewed in Table 2, for each sponge species, we listed the halogenated compounds under the correlated species. Considering the taxonomic species diagnosis of morphologic variation of spongin fibers is difficult [33], chemical composition can be used as a tool for a more accurate identification. The distribution of the halogenated compounds is widespread in *Aplysina* genre, and studies show that mainly bromoisoxazoline alkaloids have been found in almost all species. This family of metabolites was usefully employed as a chemical marker for the distinction of some taxa as *Aplysina aerophoba* and *Aplysina cavernicola,* two very physically similar species [63], but biochemically different. In another situation, majority of aerothionine was key to identify two subspecies of *A. fistularis*, which split into *A. fulva* and *A. insularis* [115].

The similarity between agelorins A and B, isolated from *Agelas oroides* and produced by *Aplysina caissara*, was essential to show the two genera, *Aplysina* and *Agelas,* have a phylogenetic relationship [76] and 11-epi-fistularin-3 was yielded by *Aplysina fulva* [98].

The presence of stereo metabolites isolated from *Aplysina* sponges as derivatives of fistularin-3 discussed by Rogers *et al.*, 2005 [98], provides evidence that enzymatic pathways are non-stereoselective in these sponges.

As can be seen, each kind of genus adds a different profile of metabolites. However, even with a different chemical profile, *Aplysina* sp. has compounds that give a clue to their evolutionary origin.

### 3.2. Bromotyramines

The substances aplyzanzine A, aplysamine-1 and aplysamine-2 present a dibromotyramine structural portion, and probably originated in accordance with Evan *et al.* [82], by amidation with other bromotyrosinated radicals. Moloka’inamine [116] and purealidin C isolated from *Psammaplysilla purea* [90] are examples of metabolites isolated from sponges of the order Verongida, having dibromotyramine in their structures. According to Carney, free phenolic groups are important precursors nitrile phenolic [96], hence the similarity between methoxylated compound, aplysamine-2, and hydroxylated analogue, psammaplin-A [84], observed by Xynas and Capon, 1989, shows that psammaplin-A may be important precursor aplysimines as much of the fistularin and its derivatives

### 3.3. Cavernicolins

**Figure 1:**
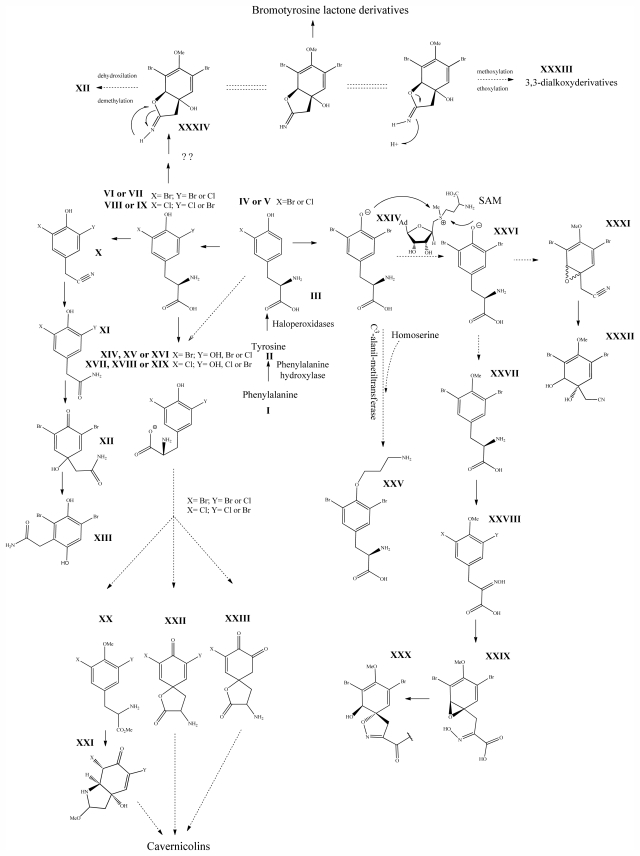
Metabolism of the bromotyrosine derived metabolites ( For the steps clarified in previous studies and For the biogenesis hypothesis).

Cavernicolines are γ and δ-lactames formed by a residual halogenated tyrosine precursor [81] and also having a bi-cyclic system. The junction of the rings occurs in carbons C-3 and C-4 in *ortho* position, while in the 7-bromocavernicolenone and in the 7-chlorocavernicolenone, this junction occurs in carbons C-2 and C-4. They can be defined as haloperoxidases with the role of converting either the 3-chloro (**V**) *ortho* 3-bromotyrosine (**IV**) in residues of 3,5-dichloro (**VIII**) or 3,5-dibromotyrosine (**VI**) or 3-chloro-5-bromotyrosine (**VII**) or 3-bromo-5-chlorotyrosine (**IX**), respectively [80]. These substances have a chiral center at C-2 and their *R* and *S* enantiomers are obtained in racemic mixtures, or relatively pure from the genus *Aplysina*. In the formation of cavernicolines, as to the substitution pattern (*ortho* or *para*) it is suggested that the biosynthesis pathway has either a halotyrosine (**XV**, **XVI**, **XVIII** and **XVIX**) or a halo-dopa (**XIV** and **XVVI**) intermediary which will form a spirolactone precursor (**XXII** and **XXIII**), allowing the formation of intermediaries in racemic or quasi-racemic mixtures. The absence of control in the absolute stereochemistry of this class is intrinsic to phenol oxidative coupling [81,117]. It is noteworthy that experimental observations [118] show that 3,5-dibromo-4-methoxyphenylalanine methyl ester (**XX**) in reaction in an anodic oxidative medium form a more appropriate intermediary (**XXI**) than the spirolactone, originating derivatives similar to the stereoisomers of the cavernicolines with considerable yields (See Figure 1).

### 3.4. Hydroverongiaquinols

The hydroverongiaquinols are 2,6-bromotyrosine phenolic derivatives. Both the hydroverongiaquinols and the verongiabenzenoids are important mediators in biosynthesis of other classes of bromotyrosine metabolites. The verongiabenzenoids are part of the biosynthesis of isoxazoline alkaloids, and hydroverongiaquinols are important precursors in the formation of metabolites which need free phenolic groups to convert themselves into α-oximine substances, such as the phenolic nitriles. However, phenolic nitriles have not been found in the genus *Aplysina*, they are found in the genus *Ianthella* where substances like the bastadins, are important chemotaxonomic markers for the genus [119].

### 3.5. Bromotyrosineketals

**Figure 2:**
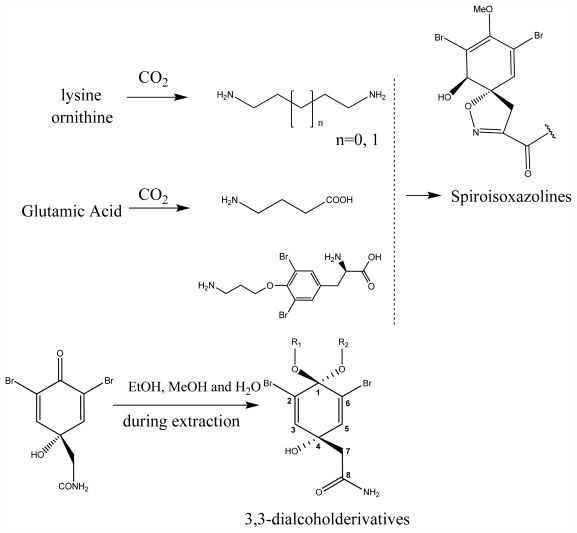
Spiroisoxazoline and acetal formation as production artefacts.

The bromotyrosineketals have a 3,5-dibromocyclohexa-2,5-dienyl ketal skeleton system. Literature shows that the dimethoxy and methoxy-ethoxy ketals (**XXXII**) isolated from *Aplysina fistularis* and from *Aplysina cauliformis* [92,93,120], are artifacts formed by the oxidation of dienone, since the dimethoxy form is obtained as a mixture of diastereoisomers [94,95], both showing antibacterial activity. Further evidence that they are artifacts is the formation of dimethoxy and methoxy-ethoxy ketals which can be explained as being formed from an arene (dienone intermediary **XII**), which suffers 1,4 additions of methanol, water or ethanol (Figure 2), and displayed a reaction described by Kasperek *et al.* [94,95,121]. However, the methoxy-butoxy and methoxy-pentoxy ketals isolated from *Aplysina thiona* [109] are not considered reaction products. Aplysinketal A was isolated only in the form of diastereoisomers, and the absence of the dimethoxy ketal indicates the non-existence of reactions during the extraction process [109]. It has been suggested that the formation of the C_4_ and C_5_ chains are formed via lysine and ornithine respectively (Figure 2) [104,114].

### 3.6. Bromotyrosine Lactone Derivatives

The bromotyrosine lactone derivatives, with the exception of aplysinimine which is an imine, are five member lactones condensated with 3,5-dibromotyrosine residues (**XXIV**). Aeroplysinin-2 is different from the others because it has a cyclodiene group instead of an aromatic ring, while aplysinadiene presents a *cis-trans* diene side chain. It is proposed that the biosynthesis of this class of substances has an imine-ether (**XXXIV**) as initial intermediary, a hydroxylated derivative of aplysinimine. This derivative will either suffer a tertiary alcohol dehydration to form aplysinimine [109,120] or will follow other biosynthetic pathways similarly to bromotyrosine lactones as aeroplysinin-2, or the verongiabenzenoids and verongiaquinols, in this case the intermediary suffers dehydroxylation and demethylation and can form artifacts such as dimethoxys and methoxy-ethoxy ketals. [122]. Aplysinolide is considered an artifact, since it possesses an α,β-unsaturated side chain which is uncommon to find linked to a lactone ring. In accordance to Cruz *et al.* [109] this substance can be formed by combining aplysinimine with Me_2_CO during the purification process (see Figure 1).

### 3.7. Oxazolidones

Oxazolidones are very common in *Aplysina*, but just two types are found: diastereoisomers of bisoxazolidone and methoxy derivative. This derivative presents two chiral centers with different stereochemistries for different species. Studies show that these bisoxazolidone’s isomers have a relative configuration of 7S*, 11R* [97] and 11R*, 7S* [106], as determined by comparison with bisoxazolidone isolated from Ascidia *Clavelina oblonga* [123] and an absolute configuration *R*, *R* [64] obtained by X-ray crystallography. There are evidences that fistularin-3 degradation promotes bisoxazolidone and aeroplysinin-1 production [13].

### 3.8. Spiroisoxazolines

The spiroisoxazolines also known as isoxazoline alkaloids form the biggest group of *Aplysina* and Verongid order metabolites. They are divided into two structure types: mono-spirocyclohexadienylisoxazolines and bis-spirocyclohexadienylisoxazolines [124].

The chemical structure of mono-spirocyclohexadienylisoxazolines (nuclei G7, G8) have essentially one spirocyclohexadienylisoxazoline ring bonded to a 1–6 carbon side chain with exception of the spiroisoxazoline acid ester (nucleus G9) [83] considered an artifact of ethanolic condensation from spiroisoxazoline acid [125]. The bis-spirocyclohexadienylisoxazolines (nuclei G1, G2) can present the same ring, which in the more common example is bonded to a 3–11 carbon side chain.

This side chain is bonded to another spirocyclohexadienylisoxazoline ring [124]. In other spiroisoxazolines, the rings suffer oxidation of the methoxy group forming a cyclohexenone (nuclei G3, G4) [74,76,101]. Cyclohexenone also suffers hydroxyl or bromine oxidations originating cyclohexenone epoxide between carbons 1 and 2 and forming oxaspirocyclohexenonylisoxazolines (nuclei G5, G6) [100]

The biosynthetic pathway for spiroisoxazolines needs tyrosine intermediates with *O*-methyl groups (**XXVII**), which are metabolized to form oxime grouped intermediates (**XXVIII**) and shortly thereafter form other intermediaries with arene oxide (**XXIX**). The nucleophilic attack of the oxime over either the epoxide or the phenol originates by breaking the epoxide which forms the isoxazole ring (**XXX**) [96]. The C_4_ or C_5_ side chains that extend out of the ring such as in aerothionin and inhomoaerothionin are produced via ornithine and lysine respectively (Figure 2) [70,94,95,126]. However, when the side chains present a 4-aminobutanoic substituent it is suspected that the amino acid involved is glutamic acid (Figure 2) [99]. In some spiroisoxazolines such as fistularin-3, and araplysillin N^9^-sulfamate, there is a 3-amino-1-propanol connector which binds itself to other structures and probably has as a precursor of its biosynthesis a decarboxylated product of the uncommon amino acid, homoserine. This amino acid is an intermediary for the enzyme S-adenosylmethionine (SAM). It is suggested that the SAM is involved in SN2 substitutions of hydroxyl of 3,5-dibromotyrosine (DBT) (**XXIV**), making it susceptible to the formation of methoxyl groups by methyltransferase and *O*-alkylated bonds via other enzymes. The enzyme responsible for *O*-alkylations is putative C^3^-alanil-methyltransferase which allows 3-amino-1-propanol connector bonding to the DBT residue, forming spiroisoxazoline residue complexes with large molecular masses (**XXV**) which are then incorporated in the isoxazoline rings [99]. The alkaloid archerine, a dimer of two imidazole rings, is probably formed by oxidative coupling [1 + 1] of two aerophobin-2 molecules [73]. In sponges of the order Verongida, the spherule cells have the capacity of stocking and secreting isoxazoline alkaloids, which are modified by enzymes located in distinct locations [127,128]. The extracts of the majority of the sponges belonging to the genus *Aplysina*, when tested, show that their enzymes convert brominated isoxazoline alkaloids into aeroplysinin-1 and dienones. Sponges of other orders are unable to perform this biotransformation [129]. Puyana *et al.* [130] demonstrated that there is no aeroplysinin-1 and dienones production when there is a decrease in the amount of spiroisoxazolines. The ecological function of this enzymatic activation is microbial pathogen growth inhibition and the repellent odor, which decreases the predatory search by fishes [129].

Although the agelocaissarines A1, A2, B1 and B2 were initially considered production artifacts as pairs of stereoisomers, this was later modified by the observation of in vitro experiments showing the absence of substances with relative stereochemistries different to those found in the work [76].

In therapeutics these substances demonstrate tumor cell cytotoxic [22], antimicrobial [15] and antihistamine [73] activity.

Spiroisoxazolines vary in different species, but also inside the single species. While *A. fulva* produces aerothionin as its major component (0.11%) [34], this same substance is not present in *A. insularis* [74] and in *A. fulva* it appears with a larger amount (0.52%) [102]. Nuñez *et al.* [97], affirms that this chemical variation may be due to either different extraction and isolation techniques, or to biological diversity of the areas in which the sponges are collected. This chemical distinction led to reinforce the hypothesis that *Aplysina aerophoba* and *Aplysina carvernicola* have metabolic differences and that *A. aerophoba*, erroneously identified in the work of Cimino *et al.* [67], was in fact *Aplysina cavernicola*.

The presence of hemifistularin, isolated together with 11-oxofistularin-3, begs us to question whether the first is a precursor of 11-oxofistularin-3 biogenesis, or a degradation product [72]. Fistularin-3 shows another type of variability. Besides having stereoisomers of (+) fistularin-3, such as (+)-isofistularin-3 and 11-epi-fistularin-3, the chemical composition of sponges contains them in irregular proportions, which makes it difficult to determine through optical rotation. In order to determine the absolute configuration of fistularin-3 and its stereoisomers, a microscale analysis with Marfey’s reagent, has been used that led to the formation of stable reaction products analyzed by LC-MS [98].

### 3.9. Verongiabenzenoids

The verongiabenzenoids are aromatic methoxylated substances which present a skeleton with a 2,6-dibromomethoxybenzene nucleus. Biogenetically methoxylated, some verongiabenzenoids can form isoxazolines [13] or epoxide intermediaries from the arene oxide, which leads to forming other verongiabenzenoids [96].

### 3.10. Verongiaquinols

The chemical structure of the verongiaquinols is either a cyclohexadien-2-one or cyclohexadien-2,6-one system, with either bromine or chlorine substituents in positions 2 and 6 and hydroxyls on carbons C-3 and C-4. They also may suffer ramifications on carbon C-4. The verongiaquinols seem to be related to degradation steps of tyrosine metabolism, as is the case of dienone (**XII**). The degradation of bromotyrosine substances such as iso-fistularin-1 and aerophobin-2 after mechanical injury led to the formation of aeroplysinin-1 (**XXXII**) and (**XII**). It is also noteworthy that the extraction of frozen sponges and consequent exposure to alkaline sea water will form dienones (**XII**) [97,130,131]. Besides being integrated at the metabolic level, dienone and aeroplysinin-1 have an important defense role for sponges: cytotoxic, algicidic, molluscicide and antibacterial activity have been reported [131,132].

Other verongiaquinols such as 2,6-dibromoquinone have been reported to inhibit the enzyme RNA polymerase II, blocking the initiation of the chain, but not its elongation [133].

It is not known for sure if (1′*R*,5′*S*,6′*S*) and (1′*R*,5′*R*,6′*S*)-2-(3′-5′-dibromo-1′-6′-dihydroxy-4′-oxocyclohex-2′-enyl)-acetonitrile are simple artifacts, as aeroplysinin-1 is able to form them in the presence of acetone by keto-enol tautomerism. However, these acetonitriles are not normally produced as metabolites of aeroplysinin-1 (**XXXII**) in other species [27].

### 3.11. Dibromocyclohexadienes

This group is comprised of two substances which present a 1,2-dihydroarene-1,2-diol and may have their biogenesis via an arene oxide (**XXXI**) in agreement with their stereochemistry [94,95]. Aeroplysinin-1 (**XXXII**) is a nitrilated substance found in dextro and levorotatory forms. The dextrorotatory isomer (+) aeroplysinin-1, has been obtained from *Aplysina aerophoba* [70], *Aiolocroia crassa, Verongula rigida, Aplysina archeri* [71] and *Psammoposilla purpurea* [134]. (−) Aeroplysinin-1 has been found in *Ianthella ardis* [135] and *Verongula gigantean* [71]. The metabolic degradation of bis-oxazolidone, isofistularin-3, aplysinamisin-1 and aerophobin-2 is known to be an important source of aeroplysinin-1 [13]. In terms of pharmacological activity, aeroplysinin-1 is a versatile substance which has demonstrated cytotoxic [134], antiprotozoal [136] and antiangiogenic [137] activities. Aplysifulvin is one of the most recently isolated substances from the sponge *A. fulva* [97]. It possesses only two methoxies and no ethoxies, and since no ethoxy derivatives were detected, the possibility that aplysifulvin is an artifact has been discarded. Hypothetically, the chemical structure of aplysifulvin suggests that the 3,3-dialkoxy ketals (with OMe and OEt groups) previously described are artifacts [97].

### 3.12. Structural Elucidation

This section describes the compilation of the ^13^C chemical shifts of halogenated compounds of the genus *Aplysina*. All compounds compiled in this review—bromotyramines (**1**–**4**), cavernicolins (**5**–**17**), hydroverongiaquinols (**18**–**20**), bromotyrosineketals (**21**–**24**), bromotyrosine lactone derivatives (**25**–**28**), oxazolidones (**29**–**32**), spiroisoxazolines (**33**–**81**), verongiabenzenoids (**82**–**86**), verongiaquinols (**87**–**99**) and dibromocyclohexadienes (**100**–**101**)—have in common 3,5-halotyrosine or halophenylalanine derivatives.

Research data shows that works from the decades of 1970, 1980 and 1990 show little or no information of ^13^C NMR as compounds (**95**–**97**) whose structural elucidation was done by mass spectrometry (MS) and ^1^H NMR spectroscopy analysis and reactions of structural identification.

The bromotyramine family skeleton can be recognized by the typical ^1^H-NMR signals, as for example, in the case of compound **1**: a singlet for aromatic protons H-2 and H-6 (δ 7.62) and four triples (δ 3.02, 2H, 8.0 Hz; δ 3.50, 2H, 8.0 Hz; δ 4.12, 2H, 5.5 Hz; δ 3.22, 2H, 5.5 Hz) attributed to H-7, H-8, H-1’, H-3’ respectively [84]. When NH-3’ has low electron density as compound **1** positively charged and compound **2** close to electrophilic oxime group, the ^13^C-NMR signals shift to downfield compared to compounds **3** and **4**,whose substituents are methyl groups [84].

Typical ^1^H NMR data from the cavernicolin class are, in the case of compound **5**, for H-3 δ 4.05 (dd, J = 10.1 Hz and J = 1.5 Hz), a singlet for H-5 at δ 7.3 and the signals for NH and OH at 6.9 (s) and 4.4 (s) respectively [83]. ^13^C NMR data shows that chlorinated carbon have their signals at downfield shifts in relation to bromine carbons as it can be seen, for example, comparing compounds **5** and **6** with **9** and compound **11** with **12**.

Biosynthetically, verongiaquinol metabolites are the oxidized form of hydroverongiaquinols, considered as a hydroquinone precursor [124,138]. Therefore, the basic difference in the ^13^C NMR between these two classes is the downfield signal of ketone carbon C-1 (δ 172.6) of compound **91** compared with hydroxylated C-1 (δ 150.0) of compound **20** [97].

Data analysis of the ^13^C NMR bromotyrosine lactone family brominated aromatic show carbon signals have more shielded signals compared to the other aromatic signals of the ring. The more is the unsaturated side branching at C-7, more deshielded are the signals of the lactone ring, with the increasing order of introducing the compounds 28, 27 and 26 [64,109].

The carbons of the spiroisoxazolinic system of most compounds with cores G_1_, G_2_, G_7_, G_8_ and G_9_ acquire values which become a standard set of values, with the exception of carbons C-2 and C4 values, which seem to be mistakenly exchanged one for another at δ 120 or δ 114, being the correct value of δ 120 for carbon C-2 due to the proximity of the hydroxyl and the C-4 for δ 114.

The mono and bis-spiroisoxazolinic ring systems could be distinguished by double ^1^H-NMR shield and deshield signals for the two rings, for example, compound **50** shows signals at δ 4.16 (1H, d, J = 8.3 Hz) for H-1 and δ 4.58 (1H, d, J = 7.9 Hz) for H 1’. A typical methylene signal at δ 4.43; 3.47 (2H, ABq, J = 18.2) is attributed to the isoxazol ring protons H-7, 7’ for this compound [29]. Today the most used techniques to elucidate absolute stereochemistry of the rings are ^1^H-NMR spectrum analysis and molecular modeling using both MM2 and MOPAC protocols of the Chem3D software [76], and also NOE-difference spectroscopy studies [100].

^13^C data reveals spiroisoxazoline ring systems have distinguished shifts. The difference between cyclohexadienone (G_1_, G_2_, G_7_, G_8_ and G_9_) and cyclohexenone (G_3_ and G_4_) systems are two chemical shifts at downfield for C-3’ and C-5’ (**54**–**55**) and one at upfield for C-2’ in G_3_ (**56**–**61**), and the same shifts for C-3, -3′, C-5, -5′, C-2, -2′ in G_4_ (**56**–**61**). The epoxide group in G_5_ and G_6_ can be characterized by the same shifts plus two differences: a strong shift at downfield for C-3 and C-3′ in G_5_ and C-3′ in G_6_. The other difference involves three chemical shifts at upfield for C-1, 1′, C-2, 2′ and C-6, 6′ of **62** and for C-1′, C-2′ and C-6′ of **63**. Some ^13^C data as δ 67.2 and δ 65.7 attributed to chiral C-11 of compound **42** still remain with its stereochemistry unsolved [76].

According to Kossuga, 2004 [123], to determine the configuration of the relative stereochemistry of bis-oxazolidone uses [α] of (7*R*,11*R*) bis-oxazolidone with absolute stereochemistry [64]. It was possible to determine the relative configuration of bis-oxazolidones (7*R,11*S) and (11*R,7*S) isolated in previous works as can be seen in Table 2 [63,65,109].

## 4. Conclusions

The genus *Aplysina* is one of the richest in secondary metabolites, which have been cataloged in 14 species from the Aplysinidae family. Most classes of compounds mentioned here present themselves brominated, and, despite the large number of species of the genus *Aplysina*, many have not been studied chemically. The halogenated compounds found in marine sponges of this genus were classified into: (A) Bromotyramines, (B) Cavernicolins, (C) Hydroverongiaquinols, (D) Bromotyrosineketals, (E) Bromotyrosine Lactone derivatives, (F) Oxazolidones, (G) Spiroisoxazolines, (H) Verongiabenzenoids, (I) Verongiaquinols and (J) Dibromocyclohexadiens.

In view of their potential for producing new compounds of pharmacological interest, sponges have been one of the most studied organisms from a chemical point of view. Over the past 20 years, hundreds of substances have been isolated from sponges, many of which have been identified and show interesting biological and pharmacological activities, as for example, antibacterial, anticoagulant, antifungal, anti-inflammatory, antimalarial, antiplatelet, antituberculosis, antiviral, immunosuppressive and neurosuppressive activities.

The species of the genus *Aplysina* also show a wide structural variety of nitrogen compounds, present only in marine sponges. Therefore they are a rich source for research of new structural models for future therapeutic applications. With the information provided in this review, we hope to facilitate research in the field and to contribute to a better understanding and knowledge of the phytochemistry of this genus.

## Figures and Tables

**Table 1:** Bioactivities of marine sponges of the *Aplysina* genus.

Activity/Species Name	Type of Extract	Bioassays Models, Organism, Dose or Route of Administration	Result	Ref.
**Antibacterial activity**				
*Aplysina archeri*	MeOH Ext.	Agar plate-*Bacillus subtilis; Escherichia coli*-1.0 mg/Disc	Active	[24]
*Aplysina fistularis*	MeOH Ext.	Agar plate-*Bacillus subtilis; Escherichia coli*	Active	[25]
	MeOH-Toluene	Agar plate-*Bacillus subtilis; Escherichia coli*	Active	[26]
	Chromatographic Fraction	Agar plate-*Staphylococcus aureus; Sarcinalutea; Klebsiella pneumonia; Proteus vulgaris; Bacteroides fragilis; Clostridium perfringens; Mycobacterium aviun*	Active	[26]
*Aplysina lacunosa*	MeOH Ext.	Agar plate-*Bacillus subtilis; Escherichia coli*	Active	[25]
	MeOH Ext.	Agar plate-*Bacillus subtilis; Escherichia coli*-1.0 mg/Disc	Active	[24]
*Aplysina laevis*	Acetone Ext.	Agar plate-*Bacillus subtilis; Escherichia coli*	Active	[27]
*Aplysina mollis*	Ether Ext.	Agar plate-*Staphylococcus aureus*-0.2μL/Disc	Active	[28]
	Ether Ext.	Agar plate-*Escherichia coli; Pseudomonas aeruginosa*-0.2 μL/Disc	Inactive	[28]
	CHCl_3_ Ext.	Agar plate-*Staphylococcus aureus*-0.2 μL/Disc	Active	[28]
	CHCl_3_ Ext.	Agar plate-*Escherichia coli; Pseudomonas aeruginosa*-0.2 μL/Disc	Inactive	[28]
	Acetone Ext.	Agar plate-*Staphylococcus aureus*-0.2 μL/Disc	Inactive	[28]
	ETOH (95%) Ext.	Agar plate-*Staphylococcus aureus; Escherichia coli; Pseudomonas aeruginosa*-0.2 μL/Disc	Inactive	[28]
	Benzene Ext.	Agar plate-*Staphylococcus aureus; Escherichia coli; Pseudomonas aeruginosa*-0.2 μL/Disc	Inactive	[28]
*Aplysina* species	Ether Ext.	Agar plate-*Staphylococcus aureus; Escherichia coli; Pseudomonas aeruginosa*-0.2 μL/Disc	Inactive	[28]
	Acetone Ext.	Agar plate-*Staphylococcus aureus; Escherichia coli; Pseudomonas aeruginosa*-0.2 μL/Disc	Inactive	[28]
	ETOH (95%) Ext.	Agar plate-*Staphylococcus aureus; Escherichia coli; Pseudomonas aeruginosa*-0.2 μL/Disc	Inactive	[28]

**Antibacterial activity**	Benzene Ext.	Agar plate-*Staphylococcus aureus; Escherichia coli; Pseudomonas aeruginosa*-0.2 μL/Disc	Inactive	[28]
*Aplysina species*	CHCl_3_ Ext.	Agar plate-*Staphylococcus aureus; Escherichia coli; Pseudomonas aeruginosa*-0.2 μL/Disc	Inactive	[28]

**Antiyeast activity**				
*Aplysina archeri*	MeOH Ext.	Agar plate-*Saccharomyces cerevisiae*-1.0 mg/Disc	Inactive	[24]
*Aplysina lacunosa*	MeOH Ext.	Agar plate-*Saccharomyces cerevisiae*-1.0 mg/Disc	Weak	[24]
			Activity	

**Antifungal activity**				
*Aplysina archeri*	MeOH Ext.	Agar plate-*Penicillium atrovenetum*-1.0 mg/Disc	Inactive	[24]
*Aplysina lacunosa*	MeOH Ext.	Agar plate-*Penicillium atrovenetum*-1.0 mg/Disc	Inactive	[24]

**Antiviral activity**				
*Aplysina archeri*	MeOH-Toluene	Cell culture-*Virus-Feline Leukemia*	Active	[29]

**Cytotoxic activity**				
*Aplysina cauliformis*	CHCl_3_-MeOH Ext. (1:1)	Cell culture-*Cells-Cho K-1*	Active	[30]
*Aplysina fistularis*	MeOH Ext.	Cell culture-*Leuk L-1210-*ED_50_ 50 mcg/mL	Active	[25]
	MeOH-Toluene	Cell culture-*CA-9KB*	Active	[26]
	Chromatographic Fraction	Cell culture-*Leuk L-1210*-IC_50_ 0.14 mcg/mL	Active	[26]
*Aplysina fulva*	Isopropanol Ext.	Cell culture-*CA*-*9KB <* ED_50_ 20 mcg/mL	Active	[31]
	Isopropanol Ext.	Cell culture-*Leuk L-1210 <* ED_50_ 20 mcg/mL	Active	[31]
	Isopropanol Ext.	Cell culture-*Leuk P-388 <* ED_50_ 20 mcg/mL	Active	[31]
*Aplysina lacunosa*	MeOH Ext.	Cell culture-*Leuk L-1210*-ED_50_ 8.2 mcg/mL	Inactive	[25]

**Hyperglycemic activity**				
*Aplysina mollis*	ETOH (95%) Ext.	Mouse-Intragastric-Dose 200 mg/kg	Active	[32]

**Table 2:** Distribution of the halogenated substances in the genus *Aplysina.*

Species	Halogenated Substances	Substance Code	Nucleus	Ref.
*A. aerophoba* (Schmidt, 1862)	Aeroplysinine 2	25	E_1_	[63]
	Aplysinadiene	26	E_1_	[64]
	(7S*,11R*)-5-[3,5-Dibromo-4-[(2-oxo-5-oxazolidinyl)]methoxyphenyl]-2-oxazolidinone	29	F_1_	[65]
	(*R*,*R*)-5[3,5-Dibromo-4-[(2-oxo-5-oxazolidinyl)] methoxyphenyl]-2-oxazolidone	31	F_1_	[64]
	Aerothionin	41	G_1_	[34]
	Homoaerothionin	47	G_2_	[66]
	Isofistularin-3	48	G_2_	[67]
	Aerophobin-1	76	G_7_	[68]
	2-(3,5-Dibromo-2-hydroxy-4-methoxyphenyl) acetamide	84	H	[69]
	3,5-Dibromo-4-methoxyphenol	85	H	[64]
	Methyl 2-(3,5-dibromo-2-hydroxy-4-methoxyphenyl) acetate	86	H	[69]
	Dibromoverongiaquinol or dienone or 3-5-dibromo-1-hydroxy-4-oxocyclohexa-2-5-diene-1-acetamide	94	I_1_	[64]
	Aeroplysinin 1	100	J	[66,70]
*A. archeri* (Higgin, 1875)	(7S*,11R*)-5-[3,5-Dibromo-4-[(2-oxo-5-oxazolidinyl)]methoxyphenyl]-2-oxazolidinone	29	F_1_	[71]
	11,19-Dideoxyfistularin 3	33	G_1_	[72]
	Archerine	43	G_1_	[73]
	Fistularin-3	46	G_2_	[29]
	11-Ketofistularin 3	51	G_2_	[29]
	Aplysina compound 1 or 1-Oxa-2-azaspiro[4,5]deca-2,6-diene-3-carboxamide, *N*,*N′*-(2-oxo-1,4-butanediyl)bis[7,9-dibromo-10-hydroxy-8-oxo, [5*R*-[5α(5′R*,9′R*,10′S*),9α,10β]]-(9CI)	57	G_4_	[74]
	Aeroplysinin 1	100	J	[74]
	(+) Aeroplysinin 1	100	J	[71]
	(−) Aeroplysinin 1	100	J	[71]
*A. caissara* (Pinheiro & Hajdu, 2001)	2-(3,5-Dibromo-4,4-dimethoxy-1-hydroxy-2,5-cyclohexadien-1-yl) acetamide	21	D_1_	[75]
	Caissarine C	42	G_1_	[76]
	Caissarine B	53	G_2_	[75]
	Agelocaissarine A1	58	G_4_	[76]
	Agelocaissarine A2	59	G_4_	[76]
	Agelocaissarine B1	60	G_4_	[76]
	Agelocaissarine B2	61	G_4_	[76]
	Caissarine A	79	G_8_	[75]
*A. cauliformis* (Carter, 1882)	2-(3,5-Dibromo-1-hydroxy-4,4-dimethoxycyclohexa-2,5-dienyl)acetamide	21	D_1_	[71]
	(7S*,11R*)-5-[3,5-Dibromo-4-[(2-oxo-5-oxazolidinyl)]methoxyphenyl]-2-oxazolidinone	29	F_1_	[71]
	11-Oxoaerothionin	52	G_2_	[77]
	Aplysinametabolite or Methyl 4-((5*S*,10*R*)-7,9-dibromo-10-hydroxy-8-methoxy-1-oxa-2-azaspiro-[4.5]deca-2,6,8-trienecarboxamido)-2-oxobutylcarbamate	64	G_7_	[78]
	Methyl 4-((5*S*,10*R*)-7,9-dibromo-10-hydroxy-8-methoxy-1-oxa-2-azaspiro-[4.5]deca-2,6,8-trienecarboxamido)-3-oxobutylcarbamate or Aplysina compound 13	65	G_7_	[78]
	Methyl-4-((5*S*,10*R*)-7,9-dibromo-10-hydroxy-8-methoxy-1-oxa-2-azaspiro[4.5]deca-2,6,8-trienecarboxamido)butylcarbamate or Aplysina metabolite 14	66	G_7_	[78]
	Aplysinamisine-1	67	G_7_	[30]
	Aplysinamisine-2	68	G_7_	[30]
	Aplysinamisine-3	69	G_7_	[30]
	Aeroplysinin 1	100	J	[78]
*A. cavernicola* (Vacelet, 1959)	Cavernicolin-1	5	B_1_	[79,80]
	5-Bromo-7α-chlorocavernicolin	7	B_2_	[81]
	5-Bromo-7β-chlorocavernicolin	8	B_2_	[81]
	7β-Bromo-5-chlorocavernicolin	9	B_2_	[81]
	7α-Bromo-5-chlorocavernicolin	10	B_2_	[81]
	Monobromocavernicolin or 5-Bromocavernicolin	11	B_2_	[81]
	5-Chlorocavernicolin	12	B_2_	[81,82]
	7-Bromocavernicolenone	13	B_3_	[82]
	7-Chlorocavernicolenone	14	B_3_	[63]
	2-(3,5-Dibromo-1-hydroxy-4,4-dimethoxycyclohexa-2,5-dienyl) acetamide	21	D_1_	[63]
	Aeroplysinine 2	25	E_1_	[63]
	11,19-Dideoxyfistularin 3	33	G_1_	[63]
	12(*R*)-Hydroxy-11-oxoaerothionin	36	G_1_	[63]
	Aerothionin	41	G_1_	[63]
	Oxohomoaerothionin	44	G_1_	[63]
	11-Deoxyfistularin-3	45	G_2_	[63]
	Homoaerothionin	47	G_2_	[63]
	Isofistularin3	48	G_2_	[63]
	11-Oxoaerothionin	52	G_2_	[63]
	(+) 3-Bromo-5-chloroverongiaquinol or (+)-3-Bromo-5-chloro-1-hydroxy-4-oxo-2,5-cyclohexadiene-1-acetamide	91	I_1_	[81]
	(+) 3-Bromoverongiaquinol or (+)-3-Bromo-1-hydroxy-4-oxo-2,5-cyclohexadiene-1-acetamide	92	I_1_	[81]
	(DL) 5-Bromoverongiaquinol	93	I_1_	[81]
	Dichloroverongiaquinol	95	I_1_	[80]
	Aeroplysinin 1	100	J	[63]
*A. conulosa* (Pulitzer-Finali, 1986)	Aeroplysinine 2	25	E_1_	[83]
*A. fistularis* (Pallas, 1766)	Aplysamine1	1	A	[84]
	Aplysamine2	2	A	[84]
	Aplysfistularine	4	A	[46]
	5-Amino-2,6-dichloro-4-hydroxycyclohex-2-enone acetic acid lactam	15	B_4_	[26]
	5-Amino-2-bromo-6-chloro-4-hydroxy-cyclohex-2-enone acetic acid lactam (5-bromo-7-chlorocavernicolin)	16	B_4_	[26]
	5-Amino-2-6-dibromo-4-hydroxy-cyclohex-2-enone acetic acid lactam or Cavernicolin	17	B_4_	[26]
	4,6-Dibromohomogentisamide	18	C	[85]
	3,5-Dibromohydroquinone-2-acetamide	19	C	[86]
	2-(3,5-Dibromo-1-hydroxy-4,4-dimethoxycyclohexa-2,5-dienyl) acetamide	21	D_1_	[87]
	Aeroplysinine 2	25	E_1_	[88]
	(7S*,11R*)-5-[3,5-Dibromo-4-[(2-oxo-5-oxazolidinyl)]methoxyphenyl]-2-oxazolidinone	29	F_1_	[71]
	Aerothionin	41	G_1_	[88,89]
	Fistularin-3	46	G_2_	[88]
	Homoaerothionin	47	G_2_	[89]
	11-Oxoaerothionin	52	G_2_	[88]
	Purealidin-L	78	G_7_	[90]
	2-(3,5-Dibromo-2-hydroxy-4-methoxyphenyl) acetamide	84	H	[26,69]
	2,6-Dibromo-1,4-benzoquinone	87	I_1_	[91]
	2,6-Dichloro-4-hydroxycyclohexa-2-5-dienone-4-acetamide	89	I_1_	[26]
	2-Bromo-6-chloro-4-hydroxycyclohexa-2,5-dienone-4-acetamide	90	I_1_	[26]
	Dibromoverongiaquinol or dienone or 3-5-dibromo-1-hydroxy-4-oxocyclohexa-2-5-diene-1-acetamide	94	I_1_	[61–65,92–96]
	Aeroplysinin 1	100	J	[96]
*A. fulva* (Pallas, 1766)	Cavernicolin-1	5	B_1_	[83,97]
	Cavernicolin-2	6	B_1_	[83,97]
	3,5-Dibromohydroquinone-2-acetamide	19	C	[34]
	2’-(3,5-Dibromo-4-hydroxyphenyl) acetamide	20	C	[97]
	2-(3,5-Dibromo-1-hydroxy-4,4-dimethoxycyclohexa-2,5-dienyl) acetamide	21	D_1_	[97]
	Aeroplysinine 2	25	E_1_	[34]
	(7S*,11R*)-5-[3,5-Dibromo-4-[(2-oxo-5-oxazolidinyl)]methoxyphenyl]-2-oxazolidinone	29	F_1_	[97]
	11-Epi-fistularin-3	34	G_1_	[98]
	11-Hydroxyfistularin-3	35	G_1_	[99]
	12(*R*)-Hydroxy-11-oxoaerothionine	36	G_1_	[34]
	12(*S*)-Hydroxy-11-oxoaerothionine	37	G_1_	[34]
	Aerothionin	41	G_1_	[34,83,97]
	Fistularin-3	46	G_2_	[31,34,83,97]
	Homoaerothionin	47	G_2_	[34]
	11-Hydroxyaerothionin	50	G_2_	[34,97]
	11-Oxoaerothionin	52	G_2_	[34,97]
	Aplysinamisine-1	67	G_7_	[97]
	Araplysillin*N*^9^-sulfamate	70	G_7_	[99]
	Fistularin-1	72	G_7_	[34]
	Fistularin-2	73	G_7_	[31]
	*N*-[5*S*,10*R*)-7,9-Dibromo-10-hydroxy-8-methoxy-1-oxa-2-azaspiro[4.5]deca-2,6,8-triene-3-carboxy]-4-aminobutanoic acid	74	G_7_	[99]
	Aerophobin-1	76	G_7_	[97]
	Aerophobin-2	77	G_7_	[97]
	Aeroplysinin 1	100	J	[34,66]
	Aplysinafulvin	101	J	[97]
*A. gerardogreeni* (Gomes & Bakus, 1992)	Aerothionin	41	G_1_	[100]
	Homoaerothionin	47	G_2_	[101]
	Aplysinone A	54	G_3_	[101]
	Aplysinone D	55	G_3_	[101]
	Aplysinone B	56	G_4_	[101]
	Calafianin	62	G_5_	[100]
	Aplysinone C	63	G_6_	[101]
	2-(3,5-dibromo-2-hydroxy-4-methoxyphenyl) acetic acid	83	H	[100]
*A. insularis* (Duchassaing & Michelotti, 1864)	5-((2,6-Dibromo-4-(2-oxooxazolidin-5-yl)-phenoxy)-methyl)-5-methoxyoxazolidin-2-one	32	F_3_	[74]
	11,19-Dideoxyfistularin 3	33	G_1_	[102]
	Aerothionin	41	G_1_	[74,103]
	Fistularin-3	46	G_2_	[74,102,103]
	Homoaerothionin	47	G_2_	[103]
	11-Dihydroaerothionin	49	G_2_	[102]
	11-Oxoaerothionin	52	G_2_	[102,103]
	Aplysina metabolite 14	66	G_7_	[74]
	14-Oxoaerophobin-2	75	G_7_	[102]
	Aerophobin-1	76	G_7_	[102]
	Aerophobin-2	77	G_7_	[102]
	(5*S*,10*R*)-Methyl 7,9-dibromo-10-hydroxy-8-methoxy-1-oxa-2-azaspiro[4.5]deca-2,6,8-triene-3-carboxylate	80	G_9_	[102]
	2-(3-Dibromo-4-hydroxyphenyl)-*N*,*N*,*N*-trimethylethanaminium	82	H	[102]
*A. lacunose* (Lamarck, 1814)	(7S*,11R*)-5-[3,5-Dibromo-4-[(2-oxo-5-oxazolidinyl)]methoxyphenyl]-2-oxazolidinone	29	F_1_	[65]
	(7R*,11S*)-5-[3,5-Dibromo-4-[(2-oxo-5-oxazolidinyl)]methoxyphenyl]-2-oxazolidinone	30	F_2_	[71]
	11,19-Dideoxyfistularin-3	33	G_1_	[104]
	Aerothionin	41	G_1_	[104]
	Fistularin-3	46	G_2_	[104]
	11-Hydroxyaerothionin	50	G_2_	[104]
	11-Oxoaerothionin	52	G_2_	[104]
*A. laevis* (Carter, 1885)	(1′*R*,5′*R*,6′*S*)-2-(3′,5′-Dibromo-1′,6′-dihydroxy-4′-oxo-cyclohex-2′-enyl) acetonitrile	98	I_2_	[27]
	(1′*R*,5’*S*,6′*S*)-2-(3′,5′-Dibromo-1′,6′-dihydroxy-4′-oxo-cyclohex-2′-enyl) acetonitrile	99	I_2_	[27]
	(+) Aeroplysinin 1	100	J	[27]
*A.* species	Aplysamine1	1	A	[84]
	Aplysamine2	2	A	[84]
	Aplyzanzine A	3	A	[105]
	2-(3,5-Dibromo-4-ethoxy-1-hydroxy-4-methoxy-2,5-cyclohexadien-1-yl)-ethanamide	22	D_2_	[71,84,106]
	Aeroplysinine 2	25	E_1_	[106]
	(7R*,11S*)-5-[3,5-Dibromo-4-[(2-oxo-5-oxazolidinyl)]methoxyphenyl]-2-oxazolidinone	30	F_2_	[71]
	(*R*,*R*)-5[3,5-Dibromo-4-[(2-oxo-5-oxazolidinyl)]methoxyphenyl]-2-oxazolidone	31	F_1_	[106]
	11,19-Dideoxyfistularin-3	33	G_1_	[72]
	11-Oxofistularin-3	38	G_1_	[72]
	19-Deoxy-11-oxofistularin	39	G_1_	[72]
	19-Deoxyfistularin-3	40	G_1_	[72]
	Aerothionin	41	G_1_	[107]
	Hemifistularin-3	71	G_7_	[72]
	(10*R*)-Ethyl-7,9-dibromo-10-hydroxy-8-methyl-1-oxa-2-azaspiro[4.5]deca-2,6,8-triene-3-carboxylate	81	G_9_	[106]
	2-(3-Bromo-4-hydroxyphenyl)-*N,N,N*-trimethylethanaminium	82	H	[108]
*A. thiona* (Laubenfels, 1950)	Aplysinketal A	23	D_1_	[109]
	Aplysinketal B	24	D_1_	[109]
	Aplysinolide	27	E_2_	[109]
	Aplysinimine	28	E_2_	[109]
	(7R*,11S*)-5-[3,5-Dibromo-4-[(2-oxo-5-oxazolidinyl)]-methoxyphenyl]-2-oxazolidinone	30	F_2_	[109]
	Aerothionin	41	G_1_	[109]
	Homoaerothionin	47	G_2_	[109]
	2-(3,5-Dibromo-2-hydroxy-4-methoxyphenyl)-acetamide	84	H	[109]
	2,6-Dibromo-4-acetamide-4-hydroxycyclohexadienone	88	I_1_	[109]
	Aplysina hydroxydienone or Dibromo compound 10	96	I_1_	[109]
	Aplysina hydroxydienoic methyl esther	97	I_1_	[109]

**Table 3:**
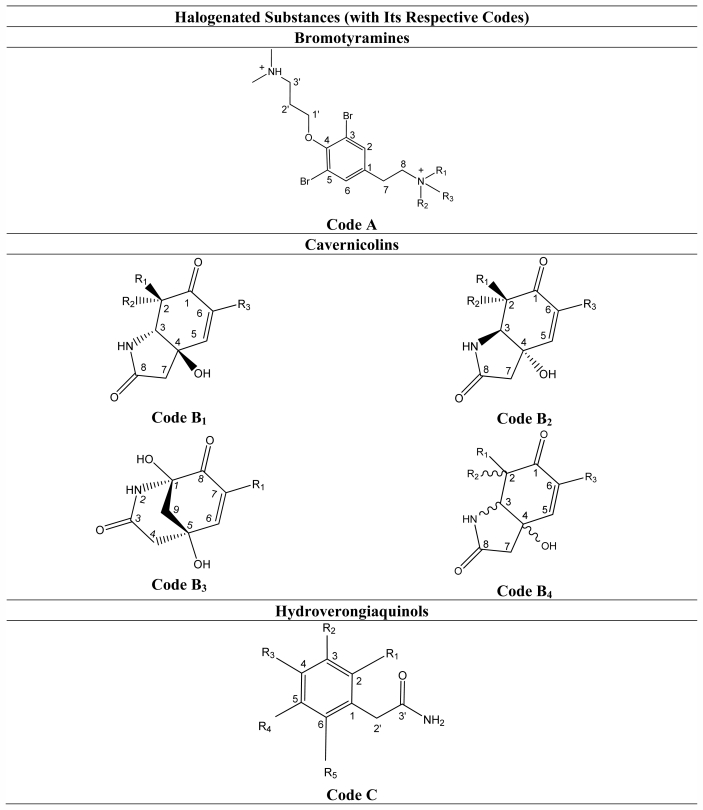

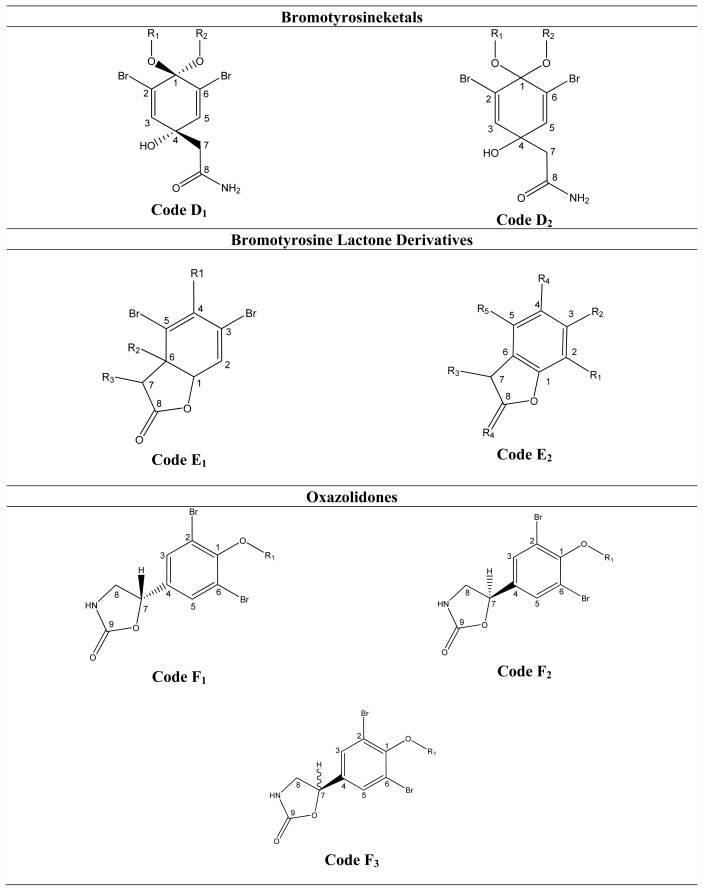

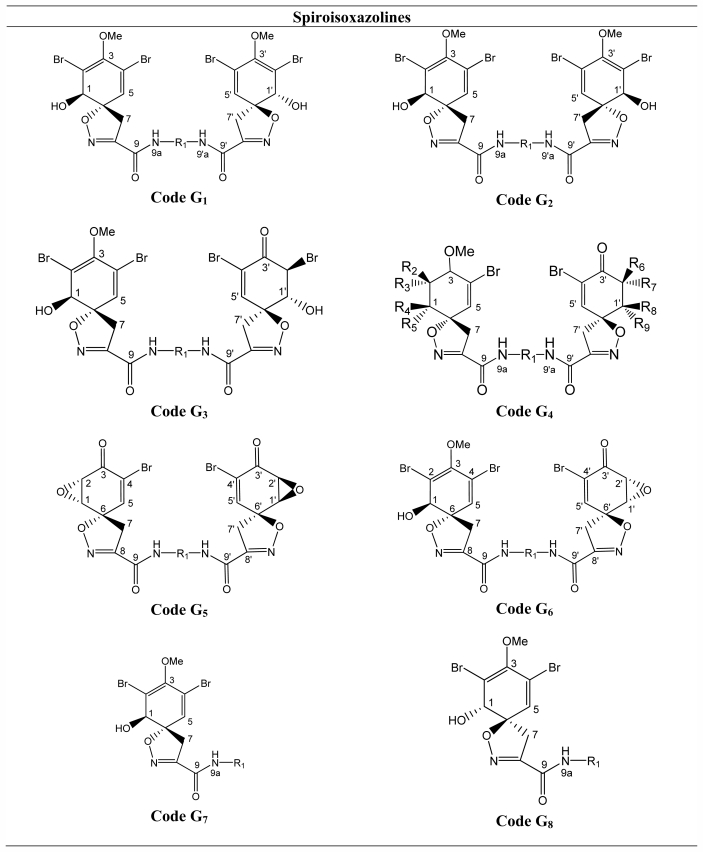

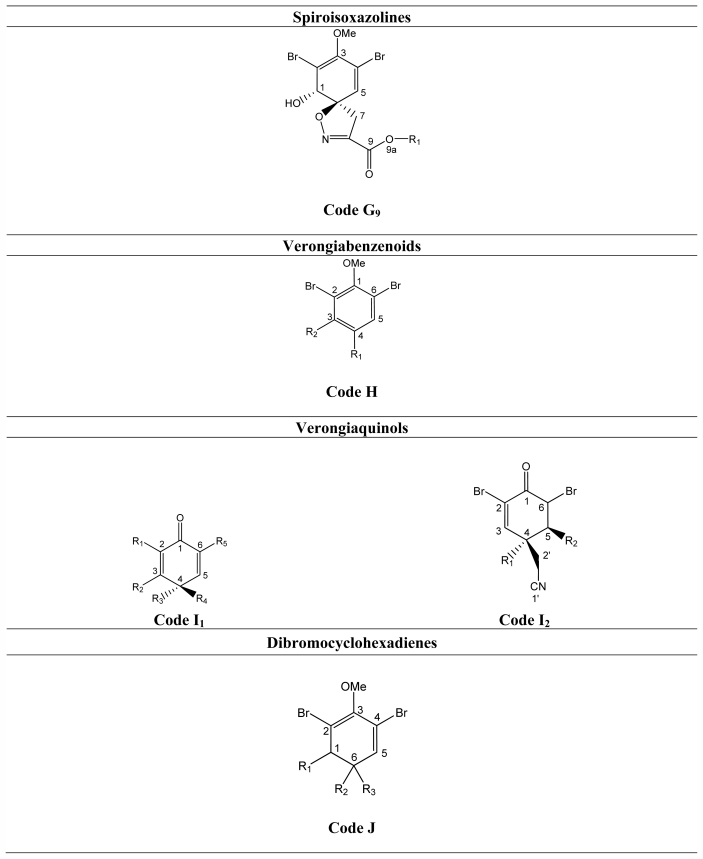
Basic skeletons of the halogenated substances isolated from sponges of the *Aplysina.*

**Table 4:**
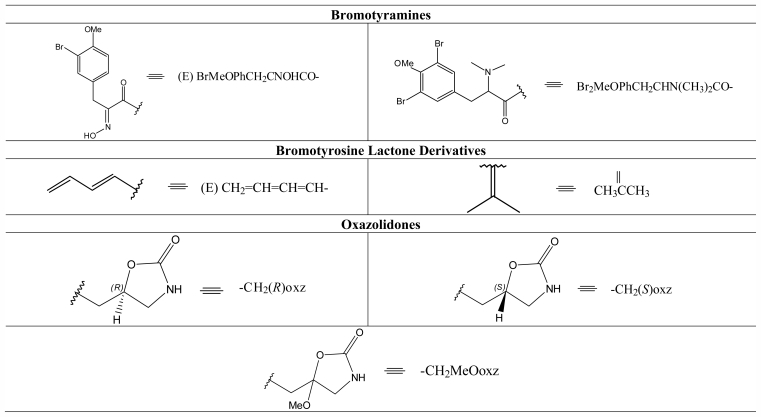

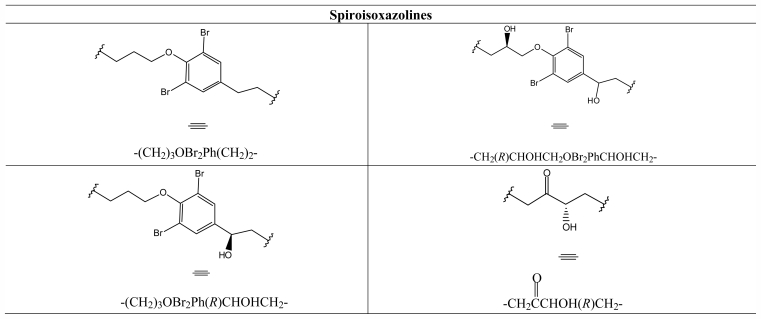

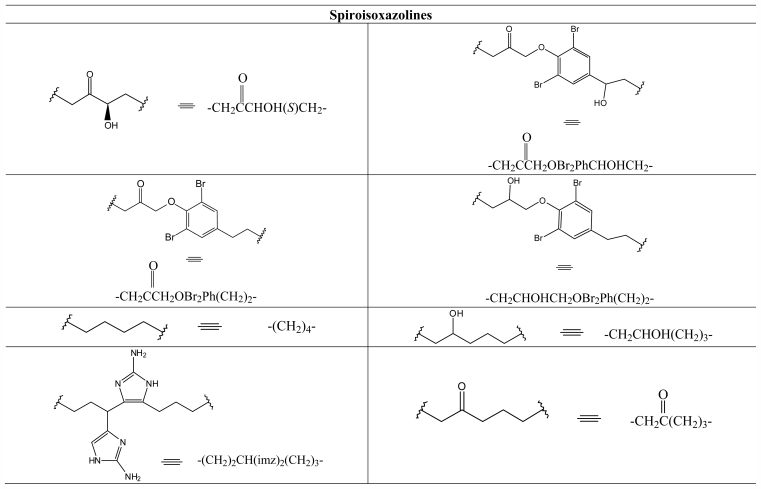

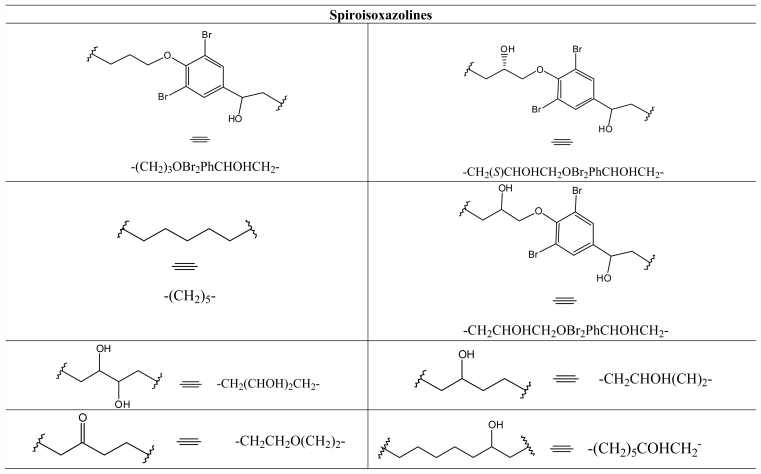

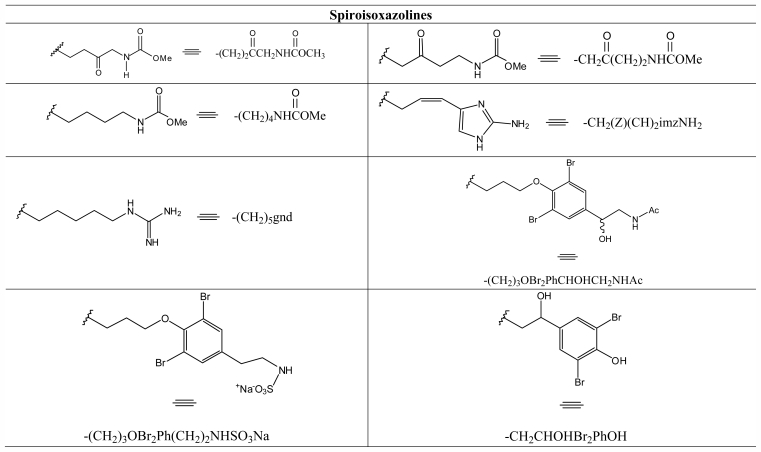

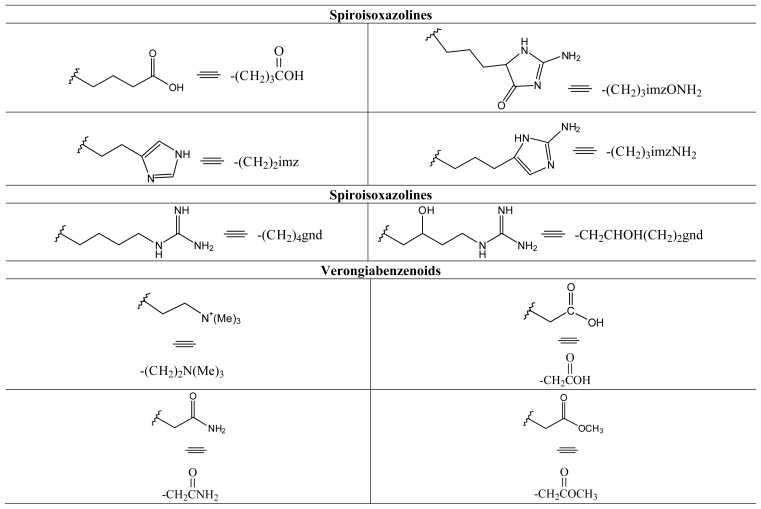

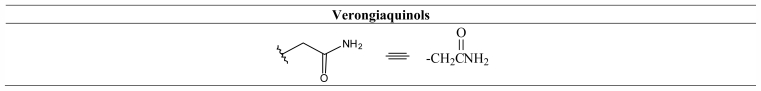
Substituent groups of the halogenated substances.

**Table 5:** Halogenated substances found in the genus *Aplysina.*

Halogen Compound	R^1^	R^2^	R^3^	R^4^	R^5^	R^6^	R^7^	R^8^	R^9^	Nucleus
1	Me	Me	-	-	-	-	-	-	-	A
2	H	(E) BrMeOPhCH_2_CNOHCO-	-	-	-	-	-	-	-	A
3	H	Br_2_MeOPhCH_2_CHN(CH_3_)_2_CO-	-	-	-	-	-	-	-	A
4	Me	Me	Me	-	-	-	-	-	-	A
5	Br	H	Br	-	-	-	-	-	-	B_1_
6	H	Br	Br	-	-	-	-	-	-	B_1_
7	Cl	H	Br	-	-	-	-	-	-	B_2_
8	H	Cl	Br	-	-	-	-	-	-	B_2_
9	Br	H	Cl	-	-	-	-	-	-	B_2_
10	H	Br	Cl	-	-	-	-	-	-	B_2_
11	H	H	Br	-	-	-	-	-	-	B_2_
12	H	H	Cl	-	-	-	-	-	-	B_2_
13	Br	-	-	-	-	-	-	-	-	B_3_
14	Cl	-	-	-	-	-	-	-	-	B_3_
15	H	Cl	Cl	-	-	-	-	-	-	B_4_
16	H	Cl	Br	-	-	-	-	-	-	B_4_
17	H	Br	Br	-	-	-	-	-	-	B_4_
18	OH	H	Br	OH	Br	-	-	-	-	C
19	Br	OH	Br	H	OH	-	-	-	-	C
20	H	Br	OH	Br	H	-	-	-	-	C
21	Me	Me	-	-	-	-	-	-	-	D_1_
22	Et	Me	-	-	-	-	-	-	-	D_2_
23	Me	Butyl	-	-	-	-	-	-	-	D_1_
24	Me	Pentyl	-	-	-	-	-	-	-	D_1_
25	MeO	OH	H	-	-	-	-	-	-	E_1_
26	H	Br	(E) CH_2_=CH=CH=CH-	OH	Br	-	-	-	-	E_1_
27	Br	OMe	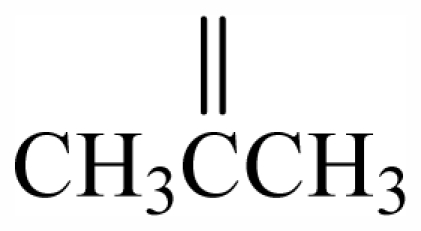	Br	H	-	-	-	-	E_2_
28	Br	OMe	H	Br	H	-	-	-	-	E_2_
29	-CH_2_(*R*)oxz	-	-	-	-	-	-	-	-	F_1_
30	-CH_2_(*S*)oxz	-	-	-	-	-	-	-	-	F_2_
31	-CH_2_(*R*)oxz	-	-	-	-	-	-	-	-	F_1_
32	-CH_2_MeOoxz	-	-	-	-	-	-	-	-	F_3_
33	-(CH_2_)_3_OBr_2_Ph(CH_2_)_2_-	-	-	-	-	-	-	-	-	G_1_
34	-CH_2_(*R*)CHOHCH_2_OBr_2_PhCHOHCH_2_-	-	-	-	-	-	-	-	-	G_1_
35	-(CH_2_)_3_OBr_2_Ph(*R*)CHOHCH_2_-	-	-	-	-	-	-	-	-	G_1_
36	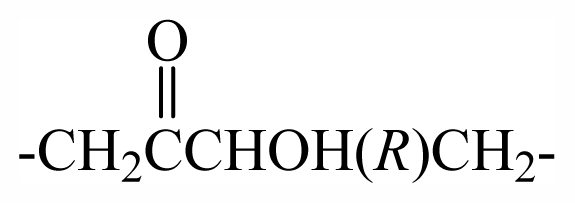	-	-	-	-	-	-	-	-	G_1_
37	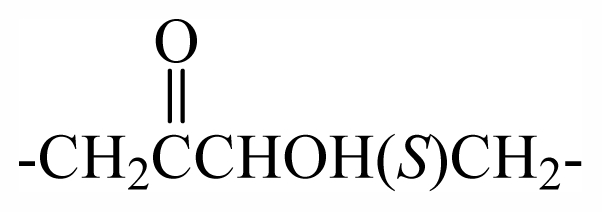	-	-	-	-	-	-	-	-	G_1_
38	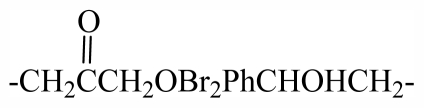	-	-	-	-	-	-	-	-	G_1_
39	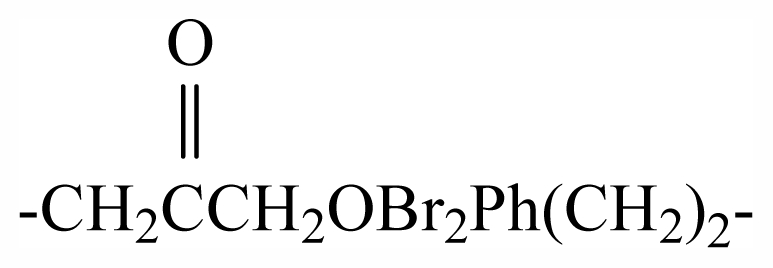	-	-	-	-	-	-	-	-	G_1_
40	-CH_2_CHOHCH_2_OBr_2_Ph(CH_2_)_2_-	-	-	-	-	-	-	-	-	G_1_
41	-(CH_2_)_4_-	-	-	-	-	-	-	-	-	G_1_
42	-CH_2_CHOH(CH_2_)_3_-	-	-	-	-	-	-	-	-	G_1_
43	-(CH_2_)_2_CH(imz)_2_(CH_2_)_3_-	-	-	-	-	-	-	-	-	G_1_
44	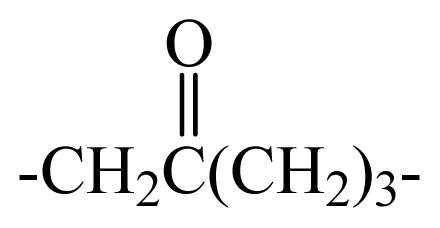	-	-	-	-	-	-	-	-	G_1_
45	-(CH_2_)_3_OBr_2_PhCHOHCH_2_-	-	-	-	-	-	-	-	-	G_2_
46	-CH_2_(*S*)CHOHCH_2_OBr_2_PhCHOHCH_2_-	-	-	-	-	-	-	-	-	G_2_
47	-(CH_2_)_5_-	-	-	-	-	-	-	-	-	G_2_
48	-CH_2_CHOHCH_2_OBr_2_PhCHOHCH_2_-	-	-	-	-	-	-	-	-	G_2_
49	-CH_2_(CHOH)_2_CH_2_-	-	-	-	-	-	-	-	-	G_2_
50	-CH_2_CHOH(CH)_2_-	-	-	-	-	-	-	-	-	G_2_
51	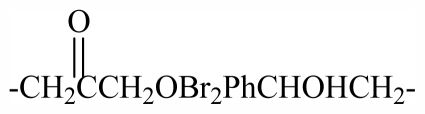	-	-	-	-	-	-	-	-	G_2_
52	-CH_2_CH_2_O(CH_2_)_2_-	-	-	-	-	-	-	-	-	G_2_
53	-(CH_2_)_5_COHCH_2_-	-	-	-	-	-	-	-	-	G_2_
54	-(CH_2_)_5_-	-	-	-	-	-	-	-	-	G_3_
55	-(CH_2_)_4_-	-	-	-	-	-	-	-	-	G_3_
56	-(CH_2_)_5_-	H	Br	OH	H	Br	H	H	OH	G_4_
57	-CH_2_CH_2_O(CH_2_)_2_-	Br	H	H	OH	H	Br	OH	H	G_4_
58	-CH_2_CHOH(CH)_2_-	H	Br	OH	H	Br	H	H	OH	G_4_
59	-CH_2_CHOH(CH)_2_-	Br	H	OH	H	H	Br	H	OH	G_4_
60	-CH_2_CHOH(CH_2_)_3_-	H	Br	OH	H	Br	H	H	OH	G_4_
61	-CH_2_CHOH(CH_2_)_3_-	Br	H	OH	H	H	Br	H	OH	G_4_
62	-(CH_2_)_4_-	-	-	-	-	-	-	-	-	G_5_
63	-(CH_2_)_5_-	-	-	-	-	-	-	-	-	G_6_
64	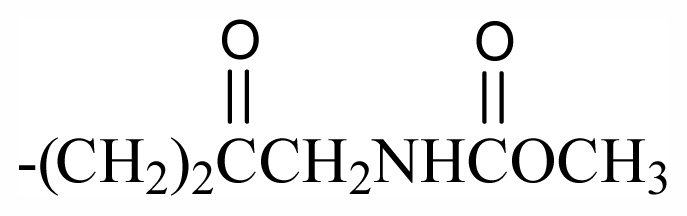	-	-	-	-	-	-	-	-	G_7_
65	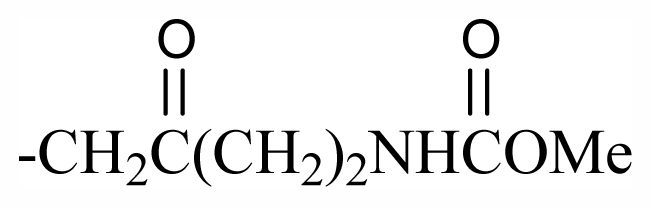	-	-	-	-	-	-	-	-	G_7_
66	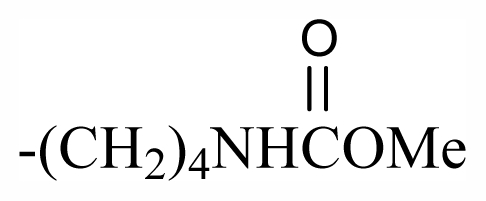	-	-	-	-	-	-	-	-	G_7_
67	-CH_2_(Z)(CH)_2_imzNH_2_	-	-	-	-	-	-	-	-	G_7_
68	-(CH_2_)_5_gnd	-	-	-	-	-	-	-	-	G_7_
69	-(CH_2_)_3_OBr_2_PhCHOHCH_2_NHAc	-	-	-	-	-	-	-	-	G_7_
70	-(CH_2_)_3_OBr_2_Ph(CH_2_)_2_NHSO_3_Na	-	-	-	-	-	-	-	-	G_7_
71	-CH_2_CHOHBr_2_PhOH	-	-	-	-	-	-	-	-	G_7_
72	-(CH_2_)_3_OBr_2_Phoxz	-	-	-	-	-	-	-	-	G_7_
73	-CH_2_Br_2_PhOoxz	-	-	-	-	-	-	-	-	G_7_
74	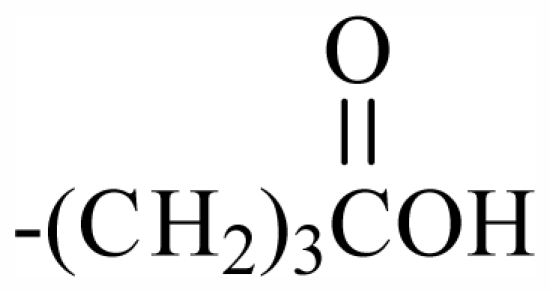	-	-	-	-	-	-	-	-	G_7_
75	-(CH_2_)_3_imzONH_2_	-	-	-	-	-	-	-	-	G_7_
76	-(CH_2_)_2_imz	-	-	-	-	-	-	-	-	G_7_
77	-(CH_2_)_3_imzNH_2_	-	-	-	-	-	-	-	-	G_7_
78	-(CH_2_)_4_gnd	-	-	-	-	-	-	-	-	G_7_
79	-CH_2_CHOH(CH_2_)_2_gnd	-	-	-	-	-	-	-	-	G_8_
80	Me	-	-	-	-	-	-	-	-	G_9_
81	Et	-	-	-	-	-	-	-	-	G_9_
82	-(CH_2_)_2_N(Me)_3_	H	-	-	-	-	-	-	-	H
83	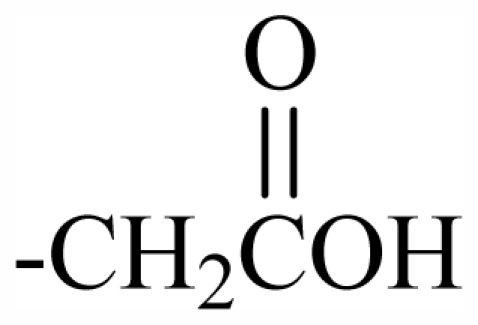	OH	-	-	-	-	-	-	-	H
84	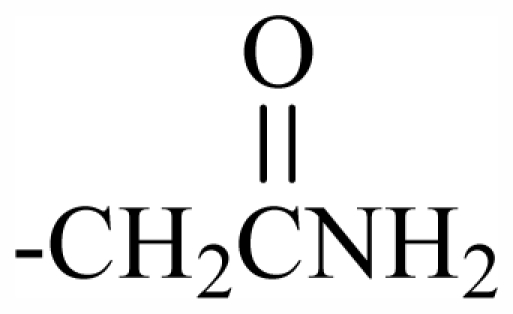	OH	-	-	-	-	-	-	-	H
85	OH	H	-	-	-	-	-	-	-	H
86	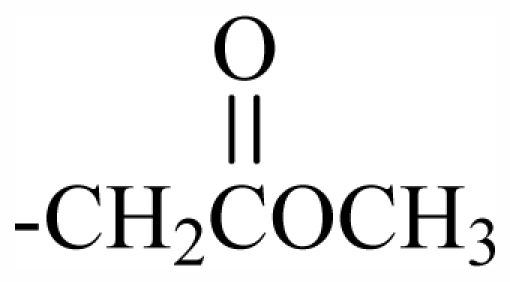	OH	-	-	-	-	-	-	-	H
87	Br	H	H	O	Br	-	-	-	-	I_1_
88	Br	H	OH	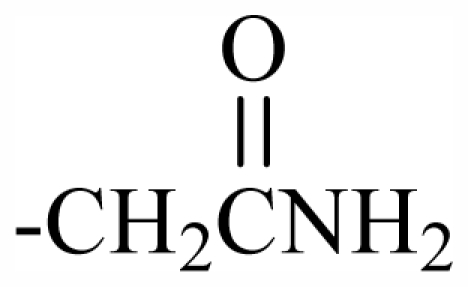	Br	-	-	-	-	I_1_
89	Cl	H	OH	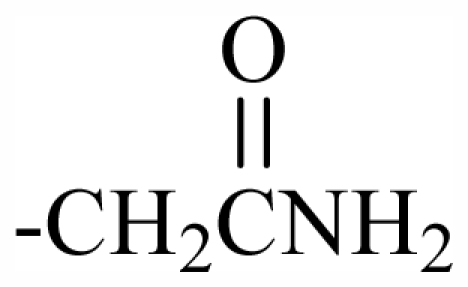	Cl	-	-	-	-	I_1_
90	Br	H	OH	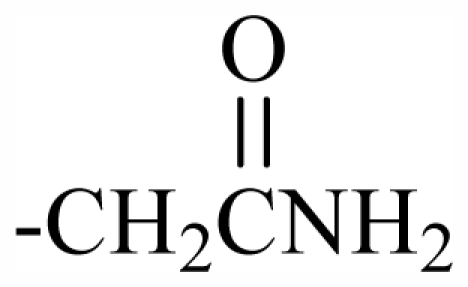	Cl	-	-	-	-	I_1_
91	Br	OH	OH	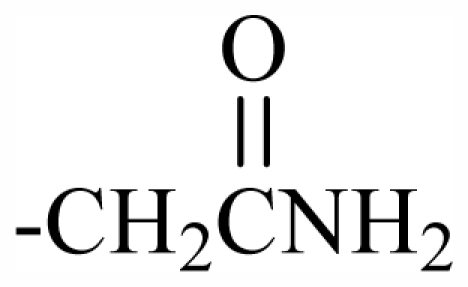	Cl	-	-	-	-	I_1_
92	Br	OH	OH	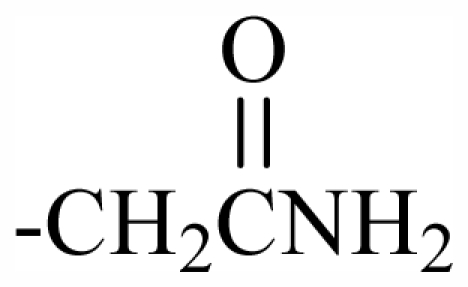	H	-	-	-	-	I_1_
93	H	OH	OH	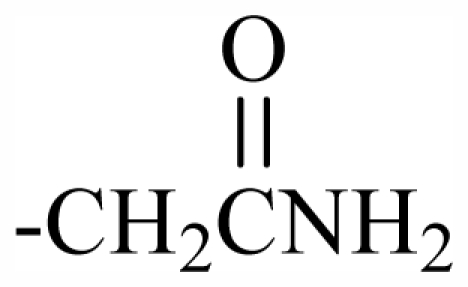	Br	-	-	-	-	I_1_
94	Br	H	OH	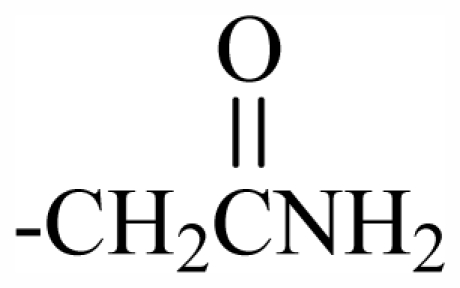	Br	-	-	-	-	I_1_
95	Cl	H	OH	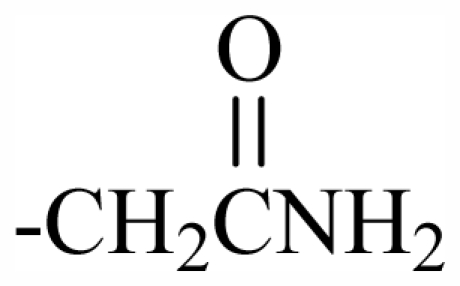	Cl	-	-	-	-	I_1_
96	Br	H	OH	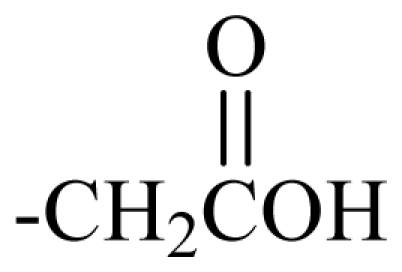	Br	-	-	-	-	I_1_
97	Br	H	O	CH_2_CH_2_OCH_3_	Br	-	-	-	-	I_1_
98	OH	OH	-	-	-	-	-	-	-	I_2_
99	OH	OH	-	-	-	-	-	-	-	I_2_
100	OH	OH	CH_2_CN	-	-	-	-	-	-	J
101	MeO	OH	CH_2_CONH_2_	-	-	-	-	-	-	J

gnd = Guanidine; imz = Imidazole; oxz = Oxazolidinone.

**Table 6:** Compilation of the ^13^C NMR data of the halogenated substances from the genus *Aplysina*. **Bromotyramines**.

Position	1	2	3	4
1	140.3	140.3	137.94	138.58
2	134.4	134.4	132.78	132.80
3	118.7	118.7	117.74	118.02
4	152.1	152.1	150.87	151.30
5	118.7	118.7	117.74	118.02
6	134.4	134.4	132.78	132.80
7	35.2	35.2	34.20	32.26
8	41.3	41.3	39.81	60.20
9	-	165.8	170.82	-
10	-	152.9	69.84	-
11	-	28.7	31.57	-
12	-	113.1	137.66	-
13	-	134.7	133.21	-
14	-	130.3	117.58	-
15	-	155.8	152.34	-
16	-	112.1	117.58	-
17	-	131.7	133.21	-
1’	71.7	71.7	69.71	71.00
2’	26.4	26.4	25.38	26.79
3’	56.9	56.9	55.41	55.77
MeO	-	56.7	60.39	-
^+^N(Me)_2_	43.7/43.6	43.7	41.51/42.92	44.06
^+^N(Me)_3_	-	-	-	44.74

**Table 7:** Compilation of the ^13^C NMR data of the halogenated substances from the genus *Aplysina*. **Cavernicolins**.

Position	5	6	9	11	12	13	14
1	184.0	184.0	183.4	188.91	188.89	80.90	82.7
2	56.9	53.1	58.0	39.78	40.08	-	-
3	68.7	64.7	67.9	58.60	58.56	168.30	171.4
4	76.6	75.7	74.4	74.15	73.41	43.1	44.0
5	149.9	149.0	146.3	150.07	145.84	69.5	69.5
6	120.8	120.8	127.4	122.45	130.18	156.90	153.2
7	43.0	45.1	42.4	43.58	43.78	118.80	129.5
8	173.9	173.9	173.6	172.65	172.72	187.8	188.9
9	-	-	-	-	-	46.6	47.6

**Table 8:** Compilation of the ^13^C NMR data of the halogenated substances from the genus *Aplysina.* **Hydroverongiaquinols**.

Position	20
1	130.0
2	133.0
3	111.0
4	150.0
5	111.0
6	133.0
2’	40.0
3’	173.4

**Table 9:** Compilation of the ^13^C NMR data of the halogenated substances from the genus *Aplysina.* **Bromotyrosineketals**.

Position	22	23
1	71.3	123.55
2	140.3	123.55
3	124.1	142.11
4	96.7	71.92
5	124.1	142.11
6	140.3	123.55
7	44.2	45.03
8	173.2	172.99
9	51.3	51.04
10	60.2	63.98
11	15.4	32.34
12	-	20.04
13	-	14.10
14	-	-

**Table 10:** Compilation of the ^13^C NMR data of the halogenated substances from the genus *Aplysina.* **Bromotyrosine Lactone Derivatives**.

Position	25	26	27	28
1	89.1	148.85	154.29	161.3
2	135.5	109.12	100.74	106.7
3	119.3	103.75	161.97	155.2
4	151.2	147.16	111.80	110.8
5	107.6	103.75	125.75	135.2
6	77.0	113.68	122.35	117.0
7	42.2	146.35	117.53	40.6
8	173.7	165.91	165.23	164.0
9	-	144.60	-	-
10	-	128.12	150.75	-
11	-	128.12	25.21	-
12	-	19.79	23.69	-
MeO	61.5	-	61.0	60.8

**Table 11:** Compilation of the ^13^C NMR data of the halogenated substances from the genus *Aplysina.* **Oxazolides**.

Position	29	30	31	32
1	153.5	151.6	142.3	140.45
2	119.3	117.6	118.2	118.63
3	131.9	130.7	130.8	131.45
4	140.6	138.9	140.0	152.60
5	131.9	130.7	130.8	131.45
6	119.3	117.6	142.3	118.63
7	76.3	74.3	54.3	75.78
8	47.9	46.9	47.0	48.42
9	159.8	158.5 or 158.2	157.8	159.05
10	75.0	73.0	53.0	72.05
11	75.5	73.5	53.55	
12	41.7	41.1	41.47	46.97
13	160.2	161.35	158.4	161.35
MeO	-	-	-	50.25

**Table 12:** Compilation of the ^13^C NMR data of the halogenated substances from the genus *Aplysina.* **Spiroisoxazolines**.

Position	33	34	35	36	37	38	39	40	41
1	75.28	75.0	75.42	75.47	75.47	75.29	75.11	75.09	75.5
2	122.07	122.0	122.74	114.14	114.14	122.11	122.11	122.10	114.2
3	148.77	148.6	149.28	149.28	149.28	148.82	148.73	148.75	149.3
4	113.81	113.7	114.16	122.78	122.78	113.85	113.85	113.78	122.7
5	132.37	132.0	132.24	132.23	132.23	132.24	132.27	132.28	133.2
6	91.67	91.8	92.48	92.64	92.64	90.04	91.95	91.56	92.6
7	40.13	39.8	40.13	40.09	40.09	39.99	39.87	40.03	40.1
8	155.24	154.9	155.27	155.12	155.12	154.80	154.79	155.14	155.5
9	160.07	160.4	161.81	161.93	161.93	160.29	160.24	160.00	161.6
1′	75.28	75.1	75.42	75.47	75.47	75.29	75.18	75.20	75.5
2′	122.07	122.1	122.74	114.14	114.14	122.11	122.11	122.10	114.2
3′	148.77	148.6	149.28	149.28	149.28	148.82	148.74	148.75	149.3
4′	113.81	113.7	114.16	122.78	122.78	113.85	113.85	113.78	122.7
5′	132.30	132.1	132.16	133.21	133.21	132.23	132.28	132.28	133.2
6′	91.67	91.8	92.48	92.54	92.54	91.86	91.59	91.76	92.6
7′	40.09	39.9	40.04	40.03	40.03	39.89	40.09	40.08	40.1
8′	155.14	155.0	154.12	154.92	154.92	155.16	155.16	155.16	155.5
9′	160.01	160.5	161.55	161.80	161.80	160.48	160.03	160.41	161.6
10	37.47	43.4	37.94	49.87	49.87	47.52	47.46	43.49	38.4
11	30.55	69.7	30.59	207.84	207.84	200.82	200.87	69.68	26.1
12	72.16	75.7	71.61	75.73	75.73	76.52	76.48	75.79	26.1
13	152.30	152.5	153.56	43.43	43.43	151.93	151.23	151.97	38.4
14	118.48	118.3	118.99	-	-	118.11	118.07	118.31	-
15	134.06	131.3	131.69	-	-	131.55	134.21	134.14	-
16	139.63	142.9	143.06	-	-	143.93	140.53	139.88	-
17	134.06	131.3	131.69	-	-	131.55	134.21	134.14	-
18	118.48	118.3	118.99	-	-	118.11	118.07	118.31	-
19	34.75	71.3	72.18	-	-	71.46	34.74	34.71	-
20	40.96	47.5	47.63	-	-	47.60	40.85	40.91	-
MeO	60.21	60.2	60.35	60.39	60.39	60.23	60.19	60.19	60.4

Position	42	43	44	45	46	49	50	51	52
1	72.4	75.41	75.50	74.60	74.73	73.55	73.8	74.67	73.55
2	119.6	114.24	120.80	121.66	121.80	120.81	121.4	121.85	120.88
3	145.9	149.29	149.31	147.92	148.06	147.15	147.7	148.06	147.13
4	111.9	122.75	114.15	115.16	115.20	113.09	113.1	115.20	113.08
5	130.1	132.30	131.32	132.15	132.36	131.25	130.6	132.30	131.20
6	89.0	92.39	92.63	91.72	91.87	90.32	91.9	91.87	90.50
7	38.5	40.26	40.21	40.27	40.30	42.53	38.7	40.30	39.70
8	153.3	155.34	155.29	155.10	155.17	154.47	153.9	155.18	154.37
9	157.4	161.44	161.81	160.05	160.52	158.98	160.0	160.46	159.11
1′	72.4	75.51	75.50	74.67	74.73	73.55	73.8	74.67	73.55
2′	119.6	114.24	120.80	121.66	121.80	120.81	121.3	121.85	20.84
3′	145.9	149.29	149.31	147.92	148.06	147.15	147.7	148.06	147.11
4′	111.9	122.75	114.15	115.16	115.20	113.09	113.1	115.20	113.08
5′	130.1	132.30	132.25	132.31	132.36	131.25	130.6	132.30	131.20
6′	88.9	92.45	92.36	91.78	91.93	90.32	91.8	91.13	90.24
7′	38.5	40.26	40.04	40.27	40.30	42.53	38.7	40.16	39.45
8′	153.4	155.41	154.93	155.23	155.25	154.47	153.9	154.77	154.05
9′	157.4	161.57	161.81	160.44	160.52	158.98	160.0	160.56	158.86
10	44.2	39.07	49.17	37.13	43.95	39.04	36.2	47.49	48.52
11	67.2 (65.7)^c^	33.92	206.18	30.37	69.47	71.04	68.0	201.34	204.43
12	30.6	33.51	37.57	71.51	76.13	71.04	45.0	76.32	38.59
13	24.0	135.87	24.11	152.27	152.29	39.02	33.6	151.22	33.82
14	35.0 (32.5)^c^	150.68	39.61	118.35	118.42	-	-	118.06	-
15	-	111.32	-	130.90	131.09	-	-	131.10	-
16	-	126.67	-	143.35	143.52	-	-	144.23	-
17	-	149.52	-	130.90	131.09	-	-	131.10	-
18	-	126.09	-	118.35	118.42	-	-	118.06	-
19	-	23.09	-	70.70	69.47	-	-	70.70	-
20	-	30.10	-	47.99	48.15	-	-	48. 01	-
21	-	40.03	-	-	-	-	-	-	-
MeO	58.4	60.44	60.38	59.75	59.86	59,63	60.0	59.86	59.63

Position	53	54	55	56	57	58	59	60	61
1	74.1	75.3	75.2	75.0	74.73	74.5	74.0	74.5	68.5
2	113.5	113.9	113.9	57.4	57.11	57.2	55.0	57.2	54.6
3	147.6	148.7	148.7	183.7	183.51	184.1	184.1	184.2	183.0
4	121.2	122.0	122.0	122.5	122.45	122.7	123.0	122.7	123.2
5	131.7	132.4	132.4	149.5	149.09	149.3	146.2	149.4	146.3
6	90.7	91.5	91.5	91.4	91.67	91.5	90.5	91.4	90.5
7	40.0	40.3	40.2	38.6	38.24	38.2	41.4	38.2	41.4
8	155.0	155.4	155.3	154.9	154.53	154.8	155.5	154.8	155.5
9	159.3	159.9	159.9	159.7	159.76	160.1	160.1	160.1	159.7
1′	74.1	75.1	75.0	75.0	74.78	74.5	74.0	74.5	68.5
2′	113.5	57.4	57.5	57.4	57.07	57.2	55.0	57.2	54.6
3′	147.6	183.7	183.7	183.7	183.51	184.1	184.1	184.2	183.0
4′	121.2	122.5	122.5	122.5	122.36	122.7	123.0	122.7	123.2
5′	131.7	149.5	149.5	149.5	149.17	149.3	146.2	149.4	146.3
6′	90.7	91.5	91.5	91.4	91.42	91.5	90.5	91.4	90.5
7′	40.0	38.6	38.6	38.6	38.10	38.2	41.4	38.2	41.4
8′	155.0	154.9	154.9	154.9	154.27	154.8	155.5	154.8	155.5
9′	159.3	159.7	159.7	159.7	159.64	160.1	160.1	160.1	159.7
10	45.7	39.7	39.5	39.7	49.17	45.9	45.9	45.9	45.9
11	67.4 and 68.9	29.7	27.5	29.7	204.70	68.8	68.8	70.3	70.3
12	32.2	24.7	27.5	24.7	39.63	34.6	34.6	32.4	32.4
13	25.5	29.7	39.5	29.7	34.78	37.0	37.0	26.0	26.0
14	36.6	39.7	-	39.7	-	-	-	37.0	37.0
15	34.4	-	-	-	-	-	-	-	-
16	39.4	-	-	-	-	-	-	-	-
MeO	60.1	60.2	60.2	-	-	-	-	-	-

Position	62	63	64	65	66	67	68	69	70
1	56.9	75.3	73.55	73.56	73.57	75.45	73.44	75.40	73.6
2	53.0	113.8	120.80	120.79	120.86	122.68	120.64	122.72	120.8
3	186.0	149.0	147.13	147.12	147.16	149.23	147.07	149.23	147.3
4	122.8	122.0	113.04	113.04	113.11	114.12	113.00	114.10	113.1
5	143.7	132.4	131.19	131.21	131.23	132.21	131.22	132.14	131.2
6	84.0	91.5	90.16	90.24	90.52	92.39	90.12	92.55	90.2
7	43.6	40.2	39.50	39.50	39.33	40.19	39.50	40.06	39.3
8	154.9	155.3	154.34	154.34	154.08	155.32	154.44	155.11	154.3
9	158.3	159.3	158.37	158.87	159.13	161.33	158.82	161.61	158.9
1′	56.9	58.1	-	-	-	-	-	-	-
2′	53.0	54.2	-	-	-	-	-	-	-
3′	186.0	186.0	-	-	-	-	-	-	-
4′	122.8	124.1	-	-	-	-	-	-	-
5′	143.7	144.1	-	-	-	-	-	-	-
6′	84.0	85.3	-	-	-	-	-	-	-
7′	43.6	44.5	-	-	-	-	-	-	-
8′	154.9	155.6	-	-	-	-	-	-	-
9′	158.3	159.9	-	-	-	-	-	-	-
10	38.6	39.8	33.83	49.89	48.58	39.49	38.53	37.86	36.2
11	26.4	29.7	38.29	205.66	205.75	123.47	28.23	30.76	29.4
12	26.4	24.7	205.66	38.29	39.75	122.33	23.28	72.30	71.2
13	38.6	29.7	49.89	33.83	35.21	130.81	27.99	153.61	150.8
14	-	39.7	156.98	156.98	157.03	151.45	40.65	118.94	117.0
15	-	-	-	-	-	118.11	157.20	131.66	133.0
16	-	-	-	-	-	-	-	142.82	140.6
17	-	-	-	-	-	-	-	131.66	133.0
18	-	-	-	-	-	-	-	118.94	117.0
19	-	-	-	-	-	-	-	71.64	33.7
20	-	-	-	-	-	-	-	47.62	44.7
21	-	-	-	-	-	-	-	173.29	-
22	-	-	-	-	-	-	-	47.62	-
23	-	-	-	-	-	-	-	22.68	-
MeO-C_3_	-	60.2	59.60	59.60	59.79	60.42	59.56	60.42	59.6
MeO-C_14_	-	-	51.54	51.54	51.28	-	-	-	-

Position	71	74	75	76	79	80	81
1	75.21	73.4	75.5	73.57	76.4	76.3	75.1
2	122.15	120.8	114.1	120.87	115.1	114.9	113.8
3	148.78	147.0	149.3	147.18	150.2	150.1	149.0
4	113.90	113.3	122.8	113.08	123.7	123.5	122.3
5	132.27	131.2	132.3	131.21	133.2	133.0	132.3
6	91.84	90.1	92.3	90.33	93.4	93.2	92.6
7	40.08	39.7	40.2	39.26	41.0	40.1	40.0
8	155.14	154.8	155.3	154.37	156.0	159.5	153.3
9	160.42	158.9	161.6	159.10	162.8	162.5	161.0
10	47.71	39.4	39.9	37.66	44.7	40.5	63.0
11	71.43	24.5	29.8	24.12	78.4	-	14.4
12	138.5	34.5	25.6	130.79	25.7	-	-
13	111.38	174.7	61.9	133.84	40.2	-	-
14	130.90	-	190.6	116.21	157.5	-	-
15	150.72	-	171.4	-	-	-	-
16	111.38	-	-	-	-	-	-
17	130.90	-	-	-	-	-	-
MeO-C_3_	60.22	59.6	60.4	59.63	61.3	59.2	60.8

**Table 13:** Compilation of the ^13^C NMR data of the halogenated substances from the genus *Aplysina.* **Verongiabenzenoids**.

Position	82	83
1	154.87	152.19
2	111.18	117.30
3	134.63	133.35
4	129.20	135.92
5	130.33	133.35
6	117.68	117.30
7	29.07	26.88
8	68.51	65.07
OMe	59.88	60.31
(Me)_3_N+	53.77	52.23

**Table 14:** Compilation of the ^13^C NMR data of the halogenated substances from the genus *Aplysina.* **Verongiaquinol**.

Position	90	97	98
1	172.6	183.0	183.0
2	119.9	122.7	123.7
3	153.2	151.7	146.6
4	70.8	75.5	74.2
5	148.8	78.4	78.9
6	127.6	56.1	57.1
1′	-	116.9	116.9
2′	-	28.4	28.4
CH_2_	45.1	-	-
CONH_2_	169.4	-	-

**Table 15:** Compilation of the ^13^C NMR data of the halogenated substances from the genus *Aplysina.* **Dibromocyclohexadiene**.

Position	101
1	86.3
2	109.1
3	149.7
4	113.6
5	140.4
6	76.5
7	42.1
8	172.8
9	60.2
10	60.3
